# Multinucleation and Polykaryon Formation is Promoted by the EhPC4 Transcription Factor in *Entamoeba histolytica*

**DOI:** 10.1038/srep19611

**Published:** 2016-01-21

**Authors:** Olga Hernández de la Cruz, Laurence A. Marchat, Nancy Guillén, Christian Weber, Itzel López Rosas, José Díaz-Chávez, Luis Herrera, Arturo Rojo-Domínguez, Esther Orozco, César López-Camarillo

**Affiliations:** 1 Universidad Autonoma de la Ciudad de Mexico, Genomics Sciences Program, Mexico City, Mexico; 2 National Polytechnic Institute, National School of Medicine and Homeopathy, Institutional Program of Molecular Biomedicine, Biotechnology Program, Mexico City, Mexico; 3 Institut Pasteur, Cellular Biology of Parasitism Unit, Paris, France; 4 INSERM U786, Paris, France; 5 National Institute of Cancerology, Carcinogenesis Laboratory, Mexico City, Mexico; 6 Metropolitan Autonomous University, Natural Sciences Department, Mexico City, Mexico; 7 Center for Research and Advanced Studies of the National Polytechnic Institute, Department of Infectomics and Molecular Pathogenesis, Mexico City, Mexico

## Abstract

*Entamoeba histolytica* is the intestinal parasite responsible for human amoebiasis that is a leading cause of death in developing countries. In this protozoan, heterogeneity in DNA content, polyploidy and genome plasticity have been associated to alterations in mechanisms controlling DNA replication and cell division. Studying the function of the transcription factor EhPC4, we unexpectedly found that it is functionally related to DNA replication, and multinucleation. Site-directed mutagenesis on the FRFPKG motif revealed that the K_127_ residue is required for efficient EhPC4 DNA-binding activity. Remarkably, overexpression of EhPC4 significantly increased cell proliferation, DNA replication and DNA content of trophozoites. A dramatically increase in cell size resulting in the formation of giant multinucleated trophozoites (polykaryon) was also found. Multinucleation event was associated to cytokinesis failure leading to abortion of ongoing cell division. Consistently, genome-wide profiling of EhPC4 overexpressing trophozoites revealed the up-regulation of genes involved in carbohydrates and nucleic acids metabolism, chromosome segregation and cytokinesis. Forced overexpression of one of these genes, EhNUDC (nuclear movement protein), led to alterations in cytokinesis and partially recapitulated the multinucleation phenotype. These data indicate for the first time that EhPC4 is associated with events related to polyploidy and genome stability in *E. histolytica*.

*Entamoeba histolytica* is the protozoan responsible for human amoebiasis, a neglected parasitic disease that causes dysentery and liver abscesses in humans^1^. This parasite exhibits some unusual features regarding cell and nuclear division in comparison with higher eukaryotes. In basal growth conditions, trophozoites can contain heterogeneous amounts of DNA. Nucleic acids can be within a single nucleus or distributed in multiple nuclei resulting in the formation of polyploidy cells^2^,^3^. This genome plasticity is the consequence of DNA duplication events without karyokinesis or cytokinesis^3^. The nuclear membrane of trophozoites remains intact throughout successive mitotic processes, which contributes to the accumulation of multiple genomes in a single nucleus^4^. Moreover, *E. histolytica* lacks the typical checkpoints that participate in surveillance mechanisms of cell division in higher eukaryotes^2^,^5^,^6^. Data mining of parasite genome confirmed the absence of known critical regulators of DNA replication and cell cycle that ensure alternation of genome duplication with chromosomes segregation in other organisms^7^. In addition, a delinking of S-phase with cytokinesis and unequal chromosomes segregation has been observed^3^,^8^. Although advances in the understanding of biological events involved in control of cell division and DNA content have been reported^6^,^7^,^8^, the regulation of these atypical cellular processes is poorly understood in this unicellular ancient eukaryote.

The human positive coactivator 4 (PC4) is a DNA-binding protein that recognizes the promoter of class II genes and facilitates the recruitment of transcriptional activators and general transcription factors stimulating pre-initiation complex assembly^9^,^10^. PC4 has additional roles in transcription termination, as well as in pre-mRNA cleavage and polyadenylation^11^. Moreover, PC4 modulates gene expression by interacting with histones H3 and H2B to mediate chromatin organization and heterochromatin gene silencing^12^,^13^. Recently, we identified an *EhPC4* orthologous gene in *E. histolytica*^14^, and showed that EhPC4 protein facilitates migration of trophozoites and destruction of epithelial colon host cells, indicating that EhPC4 has an impact on parasite virulence^15^. In this study, we further characterized the effects of overexpression of EhPC4 in trophozoites. Our results suggest that EhPC4 may represent a novel regulatory factor of genes related to multinucleation in the human pathogen *E. histolytica*.

## Results

### EhPC4 is conserved through evolutionary scale

**Figure 1:**
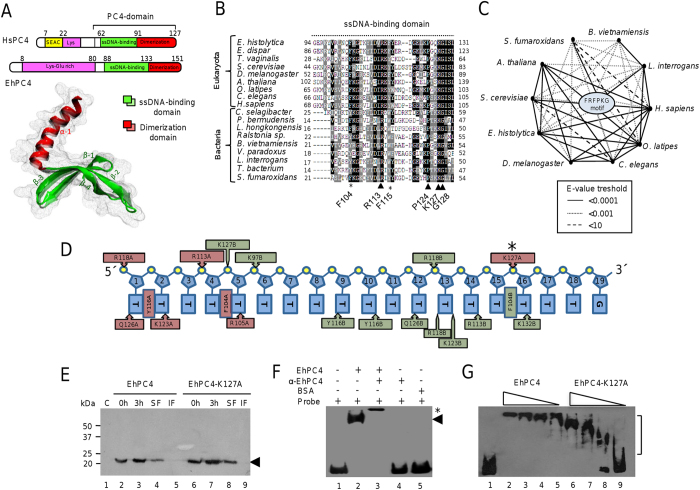
EhPC4 is an evolutionary conserved protein with DNA binding activity. (**A**) Molecular organization of human and *E. histolytica* PC4 proteins. Schematic representation of both proteins (upper panel) and superposition of human PC4-CTD (solid colors), and EhPC4-CTD (transparent colors) protein tertiary structures (bottom panel). EhPC4 3D model was deduced by homology using the structure of human PC4-CTD (PDB 1PCF) as template in Phyre program. The PDB files were used by the VMD (Visual Molecular Dynamics) viewer. Figure was drawn by O.H.C. (**B**) Multiple alignment of ssDNA binding domain from EhPC4 and representative orthologous proteins from bacteria and eukaryotes. Black boxes, identical residues; gray boxes, similar residues. Arrowheads indicate the most conserved residues in the FRFPKG motif. (**C**) Relationships between EhPC4 and orthologous proteins evaluated through PSI-BLAST analysis. The width of connecting lines indicates similarity level taking *E-value* as threshold. (**D**) Schematic representation that summarizes the more representative contacts between oligonucleotide dT_(18)_G and amino acid residues of EhPC4-CTD dimer. Red and green residues correspond to EhPC4-CTD monomer chain A and chain B, respectively. Asterisk, lysine residue (K_127_) selected for mutation. (**E**) Expression of recombinant EhPC4 and EhPC4*-*K127A proteins. Lane 1, non-transformed bacterial extract; lanes 2 and 6, non-induced bacterial extract; lanes 3 and 7, IPTG induced bacterial extract (3 h); lanes 4 and 8, soluble fraction (SF); lanes 5 and 9, insoluble fraction (IF). Arrowhead, recombinant protein. (**F**) Electrophoretic mobility shift assays (EMSA) of EhPC4 incubated with the biotin-labeled *EhADH112* promoter fragment. Lane 1, free *EhADH112* probe; lane 2, probe with EhPC4 (1 μg); lane 3, probe with EhPC4 (1 μg) and anti-EhPC4 antibody (2 μg); lane 4, probe with anti-EhPC4 antibody; lane 5, probe with BSA. Arrowhead, DNA-protein complex; asterisk, DNA-protein-EhPC4 antibody complex. (**G**) Comparison of DNA binding activity of wild-type EhPC4 and mutant EhPC4-K127A proteins through EMSA. Lane 1, free *EhADH112* probe; lanes 2 to 5, probe incubated with decreasing concentrations of wild-type EhPC4 (1.0, 0.5, 0.25 and 0.125 μg/lane, respectively); lanes 6 to 9, probe incubated with decreasing concentrations of EhPC4-K127A (1.0, 0.5, 0.25 and 0.125 μg/lane, respectively).

Bioinformatic analyzes indicated that amino acids sequence of EhPC4 contains a single-strand DNA (ssDNA) binding region and a dimerization domain in the so-called PC4 domain at the C-terminus domain (CTD). Predicted tertiary structure revealed that these conserved regions fold into four β-sheets and one α-helix, respectively, as it has been described for the human PC4 (Fig. 1A). In order to define the most conserved residues in EhPC4 domains, we performed genomic searches of orthologous proteins. Multiple alignment of the amino acid sequences of 112 PC4 orthologues from 15 proteobacteria and 97 eukaryotes including protists, algae, fungi, plants, and animals showed that the ssDNA-binding domain represents the most conserved region (Fig. 1B, Supplementary Figure S1). Phylogenetic inference analysis revealed that the bacterial PC4 domains are highly divergent in comparison with eukaryotic proteins. Indeed, they only contain the ssDNA-binding domain and lack the dimerization domain, thus being clustered in a distinct clade from their eukaryotic counterparts (Supplementary Figure S2). These data indicate that *EhPC4* gene codifies for a conserved protein that appeared early in evolution and further diversified in higher eukaryotes. Using heuristic searches and the *E-value* threshold as a similarity measure, we found that EhPC4 and orthologous proteins share a sequence located in the ssDNA-binding domain denoted here as the Fx_8_RxFx_(7–10)_Px_2_KG motif (Fig. 1C). Therefore, we investigated if this motif is potentially involved in the interaction of EhPC4 with DNA. Molecular modeling of a ternary complex composed by the EhPC4-CTD dimer bound to an oligo-dT_(18)_G predicted that F_104_, R_113_, and K_127_ residues of the FRFPKG motif interact with DNA, indicating that they may be necessary for DNA-binding affinity (Fig. 1D). The aromatic residue F_104_, could be contributing to EhPC4 DNA-binding activity via non-covalent stacking interactions with nitrogenous bases, whereas the R_113_ and K_127_ could be involved in the affinity of the protein through interactions with nitrogenous bases and DNA phosphate-backbone (Fig. 1D). *In silico* independent substitutions of these amino acid residues to alanine showed that the most significant increase in the interaction energy of ternary complex formation corresponds to the change of K_127_ residue, suggesting that this amino acid could have an important role in DNA-binding activity (Supplementary Figure S3).

### EhPC4 DNA-binding activity requires K_127_ residue

To explore if EhPC4 is a DNA-binding protein, and define the importance of the K_127_ residue in this activity, we performed site-directed mutagenesis to change the conserved K_127_ to alanine resulting in EhPC4-K127A protein. Then, we expressed and purified the recombinant wild-type EhPC4 and mutant EhPC4-K127A proteins (Fig. 1E) to compare their DNA-binding ability by electrophoretic mobility shift assays (EMSA). It has been reported that Sub1 (*S. cerevisiae* PC4 orthologue) preferentially binds to DNA sequences close to the transcription initiation site^16^, but no specific DNA-binding consensus site has been identified. Therefore an *E. histolytica* EhADH112 gene promoter fragment (−151 to +24 nt position) that contains an experimentally mapped transcription initiation site^17^ was used as probe. Results showed that wild-type EhPC4 and DNA probe form a single complex which was supershifted by the addition of specific anti-EhPC4 antibodies^15^. Control assays without EhPC4, pre-incubating reaction mixture with anti-EhPC4 antibodies or with a protein that does not have affinity for DNA (BSA), did not form any complex (Fig. 1F). Interestingly, a slight smear in electrophoretic mobility was observed when EhPC4 concentration decreases (Fig. 1G, lane 5). Remarkably, the mutant EhPC4-K127A protein exhibited a dramatic decrease in DNA-binding ability, which resulted in a more pronounced electrophoretic mobility smear at lower protein concentrations in comparison with wild-type EhPC4 (Fig. 1G, lane 9). These data indicate that the K_127_ residue is required for proper EhPC4 DNA-binding activity *in vitro*.

### EhPC4 regulates cell proliferation and DNA replication

**Figure 2:**
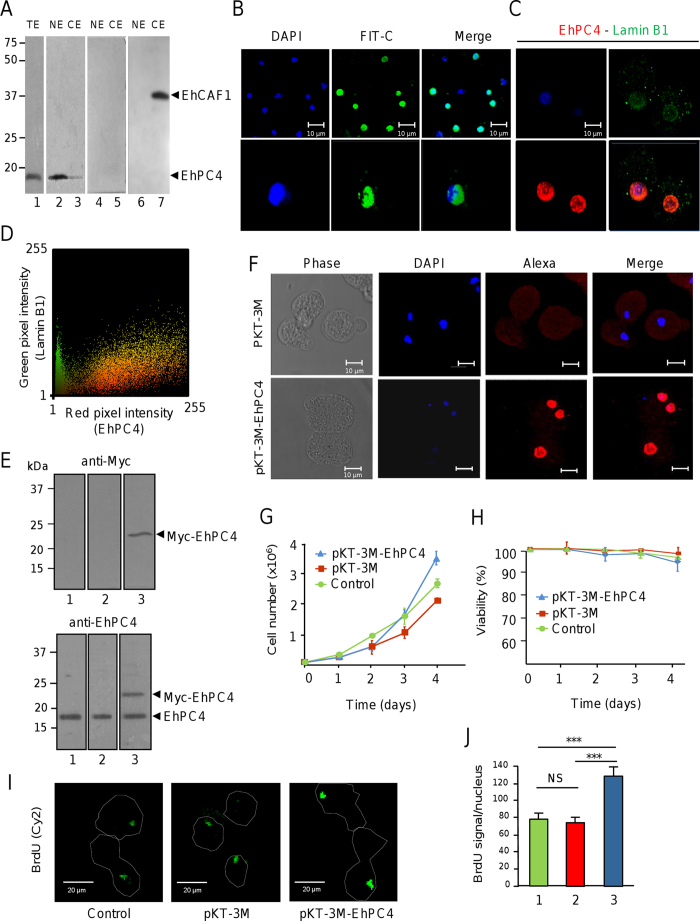
EhPC4 overexpression alters cell proliferation and DNA replication. (**A**) Immunodetection of EhPC4 in total (TE), nuclear (NE) and cytoplasmic (CE) extracts from *E. histolytica* by Western blot assays. Lanes 1 to 3, anti-EhPC4 antibodies; lanes 4 and 5, pre-immune serum; lanes 6 and 7, anti-EhCAF1 antibodies used as control. (**B**) Immunofluorescence and confocal microscopy assays showing the cellular localization of EhPC4 in trophozoites using anti-EhPC4 and FIT-C conjugated secondary antibodies. Phase contrast, DAPI stain, green channel (FIT-C) and merge are indicated. Bottom panels, single cell (100X magnification). (**C**) Coimmunolocalization of EhPC4 (Alexa 647-conjugated secondary antibodies) with lamin B1 (Alexa-488 conjugated secondary antibodies). Scale bar, 10 μm. (**D**) Colocalization analysis of EhPC4 and Lamin B proteins. Mander’s scatter plot showing the pixel intensities of EhPC4 (red) and Lamin B (green) fluorescent signals. Analysis was performed using the WCIF ImageJ software. (**E**) Immunodetection of Myc tagged-EhPC4 and endogenous EhPC4 proteins in pKT-3M-EhPC4 transfected trophozoites using anti-Myc (upper panel), and anti-EhPC4 (bottom panel) antibodies by Western blot assays. Lane 1, untransfected cells; lane 2, pKT-3M transfected cells; lane 3, pKT-3M-EhPC4 transfected cells. (**F**) Immunofluorescence and confocal microscopy assays showing the cellular localization of Myc-tagged EhPC4 using anti-Myc and Alexa 546 conjugated secondary antibodies. Phase contrast, DAPI stain, green channel (FIT-C) and merge are shown for pKT-3M (upper panels) and pKT-3M-EhPC4 (bottom panels) transfected trophozoites. (**G**) Proliferation and (**H**) viability of non-transfected, pKT-3M and pKT-3M-EhPC4 transfected trophozoites. Values shown are the mean of three independent experiments ± SD. (**I**) Confocal microscopy of fluorescent BrdU incorporation in non-transfected, pKT-3M and pKT-3M-EhPC4 transfected trophozoites. Green channel, Cy2 staining. Punctuate line, cell contour. (**J**) Quantification of fluorescent BrdU signal intensity in (**H**). In each case, 150 cells were analyzed and data correspond to the mean ± SD. Bar 1, control cells; bar 2, pKT-3M transfected trophozoites; bar 3, pKT-3M-EhPC4 transfected trophozoites.

To investigate the subcellular localization of EhPC4 in trophozoites, we analyzed fractionated protein extracts in Western blot assays. Specific anti-EhPC4 antibodies recognized the endogenous EhPC4 in total and nuclear extracts while a weak signal was observed in the cytoplasmic fraction (Fig. 2A). Cross-contamination between cellular fractions was discarded using antibodies raised against the cytoplasmic EhCAF1 deadenylase as control^18^. These results were validated by immunofluorescence and confocal microscopy assays. EhPC4 signal was found in large punctuate nuclear structures with a tendency to accumulate near to nuclear membrane, whereas a very weak signal was observed in the cytoplasm (Fig. 2B). Coimmunolocalization assays of EhPC4 with lamin B1 protein confirmed that EhPC4 is also located in the nuclear membrane (Fig. 2C). Quantitative analysis of EhPC4 and lamin B colocalization using the WCIF ImageJ software, suggested that both proteins colocalized in great extent as indicates the Pearson’s correlation coefficient (0.79) and Mander’s overlap coefficient (0.63) (Fig. 2D).

To study EhPC4 cellular functions, we first performed knock-down assays to inhibit its expression using the pEh-hyg-TetR-O-EhPC4 tetracycline inducible vector. However, the inhibition of EhPC4 was not maintained over the days even at high tetracycline concentrations. In addition, viability of trophozoites was compromised (data not shown). Therefore, we decided to overexpress the EhPC4 protein in trophozoites and investigated the biological effects through diverse cellular approaches. A Myc-tagged EhPC4 protein was produced in trophozoites via transfection of the pKT-3M-EhPC4 plasmid. Western blot assays using anti-Myc tag antibodies confirmed the overexpression of Myc-EhPC4 fusion protein in pKT-3M-EhPC4 transfectants (hereafter referred to as Myc-EhPC4 cells), but not in non-transfected and transfected with the pKT-3M empty vector cells (Fig. 2E, upper panel). These data were further validated using EhPC4 antibodies that recognized both the endogenous and Myc-tagged EhPC4 proteins (Fig. 2E, bottom panel). Using immunofluorescence and confocal microscopy assays, we showed that Myc-EhPC4 fusion protein conserves its nuclear localization (Fig. 2F). Notably, cell proliferation was significantly increased (p < 0.005) in Myc-EhPC4 cells from the third day of culture in comparison with control. Differences were even more evident at the fourth day (Fig. 2G), without significant alterations in cell viability (Fig. 2H). To investigate if differences in cell proliferation were due to an increase in DNA replication, we measured BrdU incorporation in mid-log phase trophozoites. Results showed that Myc-EhPC4 cells exhibit a significant 2-fold increase (p < 0.05) in fluorescent BrdU signal intensity in comparison with control cells indicative of an enhanced DNA replication activity (Fig. 2I,J). These differences were unlikely due to variations in the efficacy of BrdU incorporation, since the percentage of trophozoites incorporating the fluorescent nucleoside was similar in non-transfected (59%), pKT-3M (58%) and pKT-3M-EhPC4 (54%) transfected cells.

### EhPC4 overexpressing cells are multinucleated

**Figure 3:**
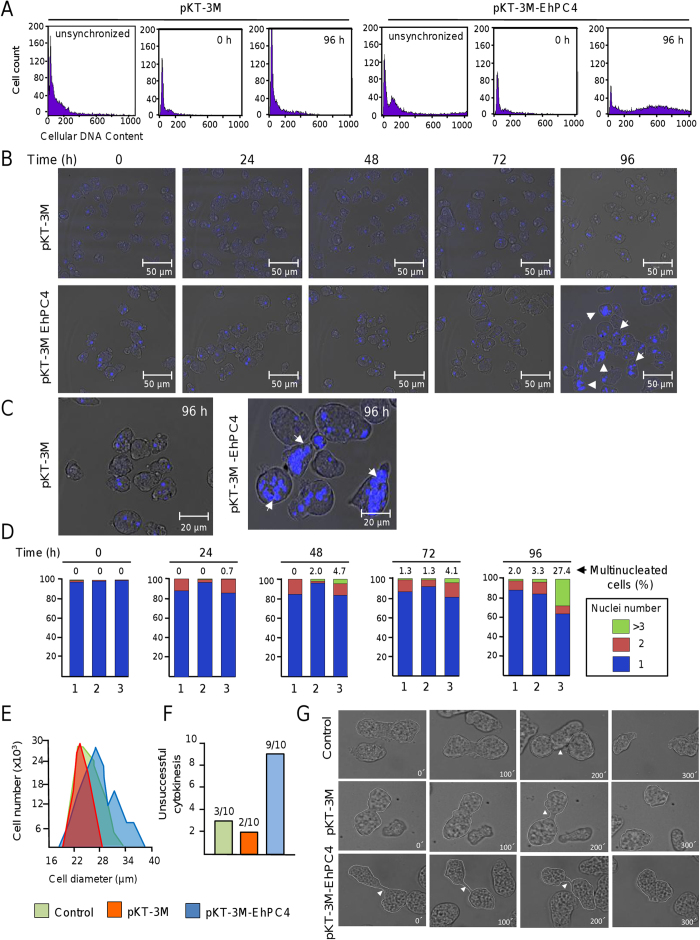
EhPC4 overexpression increases nuclei number and DNA content. (**A**) Cellular DNA content profiles obtained by FACS analysis. pKT-3M and pKT-3M-EhPC4 transfected trophozoites were synchronized, cultured for 96 h, fixed and stained with PI. The cellular DNA content (x-axis) was analyzed by flow cytometry. The y-axis represents cell count. (**B**) Fluorescence and confocal microscopy assays showing changes in morphology and nuclei number of synchronized trophozoites overexpressing EhPC4. pKT-3M and pKT-3M-EhPC4 transfected trophozoites were cultured for different time (0, 24, 48, 72 and 96 h) and stained with DAPI. (**C**) Magnification of giant multinucleated cells. Arrowhead, pleiomorphic giant multinucleated cells (polykaryon). (**D**) Number of nuclei per trophozoites from 250 randomly selected untransfected control^1^, pKT-3M^2^ and pKT-3M-EhPC4^3^ transfected cells in (**B**). (**E**) Cell diameter of untransfected (control), pKT-3M and pKT-3M-EhPC4 trophozoites at 96 h post-synchronization. (**F**) Quantification of cells with unsuccessful cytokinesis in 10 independent cell division events. (**G**) Representative time-lapse images of untransfected (control), pKT-3M and pKT-3M-EhPC4 transfected trophozoites undergoing cytokinesis. Numbers shown in the lower right corner indicate the relative passage of time in seconds. Arrowheads indicate the cytoplasmic bridge.

During our experiments, we discovered that DNA replication is augmented in Myc-EhPC4 cells. Therefore we decided to compare DNA content and nuclei number between these trophozoites and control cells. Because trophozoites contain heterogeneous amounts of DNA and nuclei number, we synchronized *E. histolytica* cultures by 12 h serum starvation to reduce DNA heterogeneity^19^. As shown in Fig. 3A, DNA content was different between pKT-3M transfected cells and Myc-EhPC4 cells. After synchronization (time 0), DNA amount was reduced, reaching similar levels in both cultures. At 96 h post-synchronization, Myc-EhPC4 cells contain a higher amount of DNA in comparison with pKT-3M control cells (Fig. 3A). Congruently, we observed the apparition of giant multinucleated Myc-EhPC4 cells, resembling a polykaryon, in a time-dependent manner (Fig. 3B). The number of cells with three or more pleomorphic nuclei (hereafter grouped and referred to as multinucleated cells) slightly increased at 48 h and 72 h post-synchronization in Myc-EhPC4 cells (4.7 and 4.1%, respectively) in comparison with non-transfected or pKT-3M transfected control trophozoites (2.0% and 1.3%, respectively). Remarkably, at 96 h post-synchronization, the number of giant multinucleated Myc-EhPC4 cells dramatically increased up to 27.4%, with up to 16 nuclei per cell (Fig. 3C,D).

These morphological alterations were accompanied by a significant increase in cell size from an initial average diameter of 23 μm in control cells to 28–37 μm in Myc-EhPC4 cells at 96 h post-synchronization (Fig. 3E). We did not detect significant differences in DNA content and nuclei number per cell when control cells were grown up to 120 h or reached 100% confluence (data not shown). Therefore, we discarded that multinucleation observed in Myc-EhPC4 cells was a secondary effect of nutrient depletion or high cell density.

The multinucleation phenotype observed in Myc-EhPC4 cells may be due to cytokinesis failure or syncytium formation by cell-cell fusion^20^. To distinguish between both possibilities, we analyzed the population of trophozoites that are undergoing cell division in the log phase of growth culture. The number of trophozoites carrying out successful cell division was estimated through live-cell imaging. Examination of living cells undergoing mitotic exit revealed that about 76% of non-transfected and 80% of pKT-3M control cells display the typical sequence of events that concludes in the formation of two daughter cells. The formation of the cleavage furrow separating the cytoplasm into two parts occurred at 100 s recording time in pKT-3M cells. Subsequently, each cell pulled in opposite directions, causing tension on constriction and forming the cytoplasmic bridge (200 s), which was cleaved to produce two daughter cells (300 s) (Fig. 3F,G; Supplementary Video 1). In contrast, only a low percentage (less than 10%) of Myc-EhPC4 cells successfully performed cytokinesis (Fig. 3F). In most trophozoites, video capture images showed the formation of the cleavage furrow (0 s to 100 s), which begins to separate and form the cytoplasmic bridge (200 s). However, after several attempts to break the bridge, most trophozoites enlarged and finally contracted again without performing cytokinesis (300 s) (Fig. 3G; Supplementary Video 2). No events indicative of cell-cell fusion were observed during our experiments.

### EhPC4 modulates genes involved in DNA metabolism, DNA replication, and chromosome segregation

**Figure 4:**
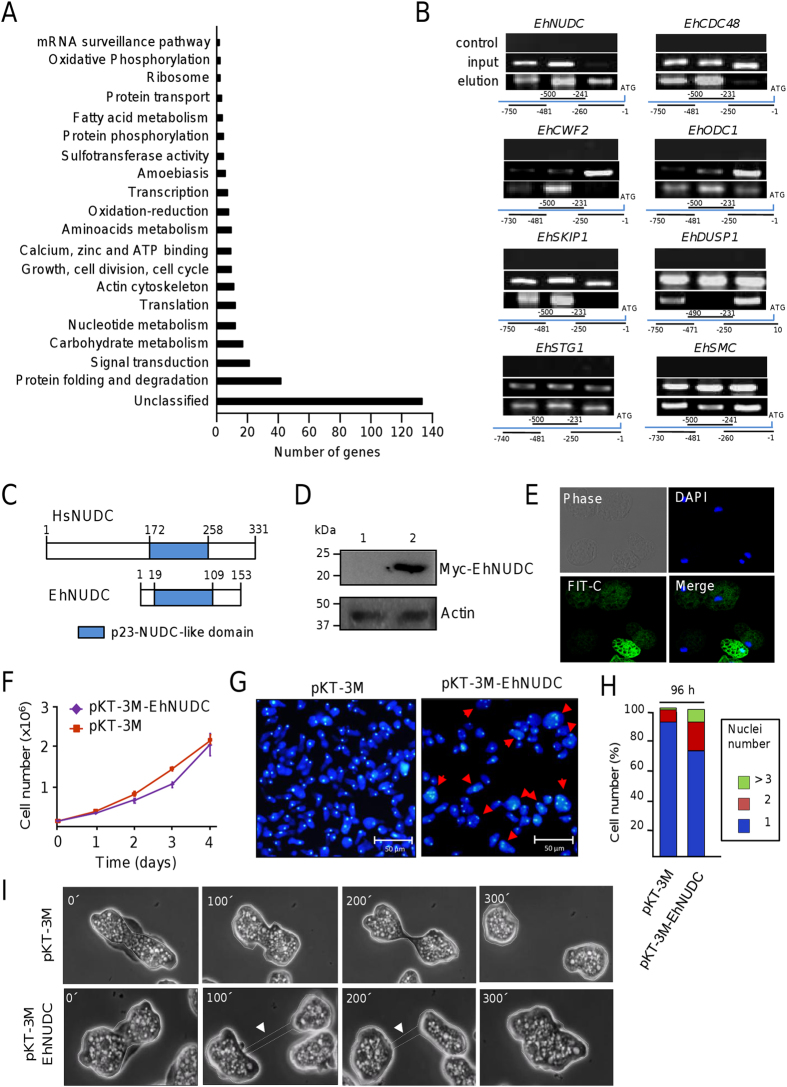
Gene modulation in Myc-EhPC4 cells and cytokinesis failure in EhNUDC overexpressing trophozoites. (**A**) Transcriptome analysis and classification of modulated genes in Myc-EhPC4 cells based in GO categories. (**B**) Chromatin immunoprecipitation (ChiP) assays. Detection of EhPC4 binding to selected gene promoters. Agarose gel electrophoresis (1%) of PCR amplicons using three different pairs of primers for each selected gene promoters. Input fraction corresponding to total sonicated DNA was used as control. Numbers in bottom scheme indicate the position of primers in promoters. (**C**) Schematic representation of human NUDC and EhNUDC proteins. The conserved P23-NUDC-like domain is indicated. (**D**) Immunodetection of Myc-EhNUDC in pKT-3M-EhNUDC transfected trophozoites using anti-Myc antibodies in Western blot assays. Lane 1, pKT-3M transfected cells (control); lane 2, pKT-3M-EhNUDC transfected cells. (**E**) Immunofluorescence and confocal microscopy assays showing the cellular localization of Myc-tagged EhNUDC in trophozoites using anti-Myc antibodies. Cells were treated with FITC-labeled secondary antibodies and counterstained with DAPI. Green channel, Myc-tagged EhNUDC; blue channel, DAPI staining. (**F**) Growth kinetics of pKT-3M-EhNUDC trophozoites. (**G**). Fluorescence and confocal microscopy assays of pKT-3M control and pKT-3M-EhNUDC transfected trophozoites stained with DAPI. Red arrowheads indicate giant multinucleated cells. (**H**) Quantification of nuclei number from 250 randomly selected EhNUDC overexpressing trophozoites. (**I**) Representative time-lapse images of untransfected (control), pKT-3M, and pKT-3M-EhNUDC transfected trophozoites undergoing cytokinesis. Numbers shown in the upper left corner indicate the relative passage of time in seconds. Arrowheads indicate the cytoplasmic bridge.

To determine the impact of EhPC4 on gene expression that can explain the cellular alterations described above, we investigated the genome-wide changes in gene expression by comparing the transcriptome of Myc-EhPC4 cells *versus* control trophozoites using *E. histolytica* DNA microarrays. Data from three biological replicates were analyzed, normalized, and raw p-values adjusted as previously described^21^. Only genes with a significant fold change (FC > 1.5; p < 0.05) were included in this analysis. Transcriptional profiling showed that 328 genes were significantly modulated (204 up-regulated and 124 down-regulated) in Myc-EhPC4 cells (Supplementary tables S1 and S2). Classification of regulated genes into functional categories according to Gene Ontology (GO) evidenced that they participate in diverse biological processes including signal transduction, cell division and metabolism, among others (Fig. 4A). Nine genes encoding *EhPGM, EhHK, EhGPI, EhALDO, EhTPI, EhGAP, EhPGK, EhENO* and *EhADH* proteins involved in glucose, fructose, and mannose utilization were significantly up-regulated. This observation suggests that an increase in carbohydrate metabolism may be supporting the requirements for the enhanced cell proliferation observed in Myc-EhPC4 cells (Supplementary Figure S4). The four *EhNDK, EhDPD, EhPNP* and *EhPAPSS2* genes whose products are involved in early reactions of nucleotide metabolism were also up-regulated (Supplementary Figure S5). These proteins may fulfill the cellular demand to sustain the increased DNA replication in Myc-EhPC4 cells. Of particular interest, a set of deregulated genes participating in DNA replication, repair, cell division, cytokinesis, and chromosome partitioning was found (Supplementary Table S3). To validate DNA microarrays data and corroborate that EhPC4 directly binds to the promoter of up-regulated genes in Myc-EhPC4 cells, we performed chromatin immunoprecipitation assays for eight selected genes (Fig. 4B). Results showed that EhPC4 interacts with the core promoter of *EhNUDC, EhCDC48, EhSTG1, EhSMC, EhCWF2, EhODC1, EhSKIP1* and *EhDUSP1* genes *in vivo* suggesting that these genes are true EhPC4 targets.

### EhNUDC alters cytokinesis and recapitulates the multinucleation event

To further obtain mechanistic insights about the EhPC4-mediated multinucleation event, we focused in the study of EhNUDC (nuclear movement protein) whose expression was 2.7-fold up-regulated in Myc-EhPC4 cells. The predicted EhNUDC amino acids sequence (C4M7J1) reported in AmoebaDB (http://amoebadb.org/amoeba/) contains the characteristic p23-NUDC-like domain and shows 42% identity with the human NUDC homologue (Q9Y266) involved in cytokinesis, nuclear migration and mitosis (Fig. 4C). In order to investigate the potential role of EhNUDC in multinucleation in *E. histolytica*, a Myc-tagged EhNUDC construct was ectopically expressed in trophozoites, and the number of nuclei per cell was determined as described above. Results of Western blot and immunofluorescence assays confirmed the expression of Myc-EhNUDC protein in the cytoplasm of pKT-3M-EhNUDC transfectants (Fig. 4D,E). Cell proliferation of pKT-3M-EhNUDC cells was not significantly affected at the fourth day in comparison with pKT-3M control (Fig. 4F). Notably, EhNUDC overexpressing trophozoites appeared as larger cells with irregular contours, and alterations in morphology accompanied with the formation of cells clumps. The proportion of flat cells containing multiple nuclei was significant higher in Myc-EhNUDC cells in comparison with control cells (Fig. 4G,H). To define if multinucleation also results from cytokinesis failure, we followed cell divisions of pKT-3M control and pKT-3M-EhNUDC transfected cells by live-cell imaging. Examination of living cells undergoing mitotic exit revealed that 79% of control trophozoites display the typical sequence of events that concludes in the formation of two daughter cells. In contrast, only a reduced number of Myc-EhNUDC trophozoites (30%) carried out successful cytokinesis, and cell division attempts of most trophozoites resulted in polykaryon formation (Fig. 4I; Supplementary Video S3). No evidence of cell-cell fusion events was observed. These results indicate that multinucleated Myc-EhNUDC trophozoites were originated from cytokinesis failure.

## Discussion

Genetic stability is achieved by the coordinated regulation of DNA replication, chromosomal segregation, and cell cycle checkpoints in almost all eukaryotic lineages. In human cells, abnormal karyotype with multiple structural and numerical aberrations of chromosomes leads to multinucleation, polyploidization, aneuploidy and cancer^22^. In protozoan parasites, multinucleation and polyploidy are frequently seen in culture. However, the molecular mechanisms underlying these cellular events and their functional relevance are poorly understood. In *E. histolytica* trophozoites multinucleation and polyploidy contribute to their genetic diversity and allows them to develop specific responses to environment and host challenges. It may also provide a better adaptation to host conditions during tissue invasion in acute disease^23^,^24^,^25^,^26^. However, the operating mechanisms of polyploidy and multinucleation are not clear and most regulating molecules have not been identified yet. Therefore, the early branching eukaryote *E. histolytica* represents a suitable model for genome plasticity studies.

In this investigation we showed that EhPC4, the positive coactivator 4 of *E. histolytica*, is a *bona fide* DNA-binding protein *in vitro*. Notably, the inability of the mutant EhPC4-K127A protein to properly interact with DNA highlights the relevance of the novel FRFPKG motif for EhPC4 DNA affinity. In addition, the smears observed in EMSA at the lower protein concentrations are typical of DNA-binding proteins with a non-specific binding sequence^27^. These observations strengthened the hypothesis that EhPC4 is a non-specific DNA-binding protein, which may be recruited by additional transcription factors to target promoters, as it has been described for homologous proteins in other organisms^28^. For example, the human PC4 participates in diverse processes through its non-specific binding to DNA. During the early stages of DNA double-strand breaks repair, PC4 recognizes DNA rupture sites across the genome and facilitates the subsequent steps of DNA repair^29^. During transcription initiation, Sub1 (*S. cerevisiae* PC4 orthologue) binds to the promoter region of specific genes and plays both a negative and a positive role in transcription initiation and elongation. Upon promoter melting, Sub1 interacts with the DNA template, stabilizing the open complex and stimulating gene transcription^29^. In addition, Sub1 has DNA-independent functions when it associates with Spt5 to influence RNA polymerase II transcription elongation rate^30^.

Here, we used various cellular and molecular approaches to dissect the molecular functions of EhPC4. A significant increase in cell proliferation, cell size, and DNA replication was found in parasites overexpressing EhPC4, resulting in the formation of giant multinucleated trophozoites. Interestingly, these morphological changes may be linked to virulence since EhPC4 overexpressing trophozoites display exacerbated virulence properties represented by a significant increase in trophozoites migration and destruction of epithelial colon host cells^15^. A similar association between pathogenicity, cell size and polyploidy has been reported in fungi. During infection, *Cryptococcus neoformans* exhibits cellular enlargement *in vivo*, producing cells up to 100 μm large that are polyploid and uninucleated (namely Titan cells). The higher DNA content may be the due to increased DNA replication. The increase in cell size was related to a reduced phagocytosis by host mononuclear cells, and an increased resistance to oxidative and nitrosative stress, which results in an increased pathogenicity^31^. On the other hand, polykaryon formation has been found in cells following multinucleation. In *E. histolytica*, an early report suggested that these structures might be formed in cell culture^32^. However, their biological significance is unknown, and they still represent an unsolved issue in protozoan parasites. We also observed that Myc-EhPC4 trophozoites undergoes polyploidy evidenced by giant nuclei, followed by production of polymorphic polykaryon of different nuclear size. Our data indicate that these events were associated to asynchronous rates of nuclear and cytoplasmic divisions in response to EhPC4 overexpression.

Along with the need to sustain a larger cell, the morphological changes observed in Myc-EhPC4 trophozoites require the activation of a precise genetic program in trophozoites. Indeed, DNA microarrays profiling revealed the modulation of genes involved in cell proliferation, carbohydrates and nucleotides metabolism, DNA replication, cytokinesis, and chromosome partitioning among others. The coordinated function of these genes supports the phenotype observed in Myc-EhPC4 trophozoites. Expression profiling, chromatin immunoprecipitation, and cellular approaches identified EhNUDC as a novel EhPC4 target in *E. histolytica*. Interestingly, ectopic overexpression of EhNUDC partially recapitulated the multinucleation phenotype. NUDC is a dynein-associated nuclear movement protein that regulates microtubule organization during mitosis and cytokinesis in mammalian cells^33^. In *Aspergillus nidulans*, NUDC is needed for nuclear movement; it is part of the dynein/NUDF complex that regulates microtubule organization and spindle formation^34^. Mammalian NUDC is essential for cell proliferation in both normal and tumor tissues; its expression increases in various cellular types undergoing mitosis or stimulated to proliferate^35^. Notably, it was shown that overexpression of human NUDC induces cytokinesis failure and multinucleation^33^. Intriguingly, down-regulation of NUDC also affects mitosis and induces the formation of multinucleated cells. This phenotype is reversed by ectopic expression of wild-type NUDC, but not by NUDC with mutations in the Polo-like kinase 1 (Plk1) phosphorylation sites, which suggests that Plk1 phosphorylation of NUDC may influence cytokinesis^36^. Interestingly, a Plk1 orthologous gene was found overexpressed in Myc-EhPC4 trophozoites, which suggests that EhNUDC could also be regulated by Plk1-mediated phosphorylation during cytokinesis in *E. histolytica*. These observations deserve further investigation. It is important to note that the multinucleation events observed here are specific of EhPC4 and EhNUDC overexpression, as the overexpression of the EhCAF1 deadenylase did not produce the same phenotype (our unpublished data).

In conclusion, our results suggested for the first time an unexpected role for the transcription factor PC4 in the regulation of genetic stability in a unicellular eukaryote. They also suggested that mechanisms contributing to polyploidy in eukaryotes were originated from early branching eukaryotes and further diversified in higher eukaryotes. These data may be useful for the search for new targets to abrogate cell proliferation and survival in this human pathogen.

## Methods

### Cell cultures

*E. histolytica* trophozoites (HM1:IMSS strain) were axenically grown at 37 °C in Diamond’s TYI-S-33 medium and harvested during exponential growth phase for experiments.

### *In silico* analysis

PC4 orthologues were identified by BLAST in NCBI database using the EhPC4 amino acid sequence (UNIPROT: C4M1H2) as query. Functional domains were predicted using MyHits server. Multiple alignments were performed using the ClustalW program. The phylogenetic tree was constructed by the Neighbor-Joining method using the MEGA software (version 5.0). EhPC4 tertiary structure was predicted using the Phyre program and the construction of structural alignment was performed using VMD viewer. Molecular modeling of CTD-EhPC4 dimer in complex with a 19-mer oligonucleotide (oligo-dT_(18)_G) was generated by homology using the MOE program. Amino acids candidate for mutation were identified according to their relevance for DNA-protein interaction based on molecular modeling.

### Plasmids construction

*EhPC4* (EHI_192520) and mutated *EhPC4-K127A* genes were cloned into the pRSET-A expression vector following standard protocols; *EhPC4* and *EhNUDC* (EHI_023890) genes were cloned into the pKT-3M vector^37^.

### Expression and purification of recombinant proteins

Recombinant EhPC4 and EhPC4-K127A proteins were expressed adding 1 mM IPTG for 3 h in *E. coli* BL21(DE3)pLysS cells that were previously transformed with pRSET-EhPC4 and pRSET-EhPC4-K127A plasmids, respectively. They were purified under native conditions by affinity chromatography using Ni Sepharose^TM^ High Performance His trap^TM^ HP (GE Healthcare) columns.

### Western blot assays

Nuclear and cytoplasmic extracts of *E. histolytica* trophozoites were obtained as described^38^. Protein concentration was measured by the Bradford method. Proteins (50 μg per lane) were separated by 12% SDS-PAGE and electrotransferred to 0.2 μm nitrocellulose membrane (BIO-RAD) using standard methods. Endogenous EhPC4 and ectopically expressed Myc-tagged EhPC4 proteins were detected using anti-EhPC4 polyclonal antibody (1:2000)^15^. Myc-EhPC4 and Myc-EhNUDC were detected using Myc-Tag antibodies (1:2000; Cell Signaling).

### DNA binding assays

Electrophoretic mobility shift assays (EMSA) were performed using the *EhADH112* gene promoter fragment (−151 to +24 nt) as probe^17^. The probe was biotin-labeled using the Biotin 3′ End DNA Labeling Kit (Thermo Scientific Pierce) and incubated with different amounts of recombinant EhPC4 and EhPC4-K127A proteins. For supershift assays, specific anti-EhPC4 antibody (1 μg) was added. DNA-protein complexes were resolved through 6% non-denaturing PAGE, transferred to Hybond^TM^-N+ membrane (GE Healthcare) and revealed using the Chemiluminescent Nucleic Acid detection module™ kit (Thermo Scientific Pierce).

### Immunofluorescence and laser confocal microscopy

Immunofluorescence assays were performed as previously described^18^. Immunostaining was carried out with anti-EhPC4 (1:200) or Myc-tag (1:200) antibodies followed by incubation with anti-rabbit FITC antibodies (1:200; Jackson ImmunoResearch). DNA was counterstained with 2.5 μg/ml DAPI (Invitrogen) for 1 min. For coimmunolocalization studies staining was carried out with anti-EhPC4 (1:100) and anti-lamin B1 (Abcam ab16048; 1:100) antibodies, followed by incubation with anti-rabbit Alexa 647 (Invitrogen; 1:200) and anti-mouse Alexa 488 (Invitrogen; 1:200), respectively. Light optical sections were obtained through a Nikon inverted microscope attached to a laser confocal scanning system (TCS SP2, Leica Microsystems) and analyzed using the Confocal Assistant software Image J. A three-laser beam (350, 488 and 568 nm) was used for excitation of the fluorophores, using a 25 Plan-NEOFLUAR oil immersion lens (0.8 numerical aperture). Immunofluorescence staining experiments were done by triplicate and multiple serial sections were examined. Secondary antibodies alone were used as negative controls. EhPC4 and lamin B1 colocalization was analyzed from entire confocal microscope images using WCIF ImageJ. Background was corrected using the background subtraction function. Pearson’s Correlation coefficient and Mander’s Overlap coefficient were calculated with the plugin intensity correlation analysis.

#### Transfection assays

Trophozoites were transfected with pKT-3M-EhPC4, pKT-3M-EhNUDC or pKT-3M plasmids by electroporation as previously described^39^. The pKT-3M-EhPC4 constructs contains the 5′ and 3′ downstream regions of cysteine synthase gene, and the neomycin resistance gene^37^. Two subsequent pulses were applied using a Gene Pulser apparatus (BIO-RAD), with an exponential discharge of 1200 V/cm and a capacitance of 25 μF. Drug selection of transfectants was performed by gradually increasing the G418 concentration up to 60 μg/ml.

#### Cell proliferation, cell viability and cell size determinations

1.5 × 10^5^ trophozoites were inoculated into culture tubes with TYI-S-33 medium and grown at 37 °C during 24, 48, 72 and 96 h. At each time, cell number, cell diameter and viability were measured using the TC20^TM^ Automated Cell Counting System (BIO-RAD) and the TC10 trypan blue dye method as described by the manufacturer. Data were expressed as the mean ± standard deviation (SD) of three independent experiments.

#### DNA replication assays

DNA replication experiments were performed using the DNA Replication Assay kit (Upstate Biotechnology) according to manufacturer’s recommendations. Trophozoites incorporating the fluorescent BrDU in DNA were examined through a Nikon inverted microscope attached to a laser confocal scanning system (TCS SP2, Leica Microsystems) and analyzed with Image J software. Fluorescence intensity of 250 randomly selected cells was measured with the Leica confocal software. Three experiments were performed and data were expressed as the mean fluorescence ± SD.

### Determination of nuclei number

Trophozoites were synchronized by serum starvation for 12 h as described^3^; 1.5 × 10^5^ cells were inoculated into culture tubes with TYI-S-33 complete medium and grown at 37 °C for 24, 48, 72 and 96 h. Then, trophozoites were fixed with 4% paraformaldehyde on sterile coverslips, nuclei were stained with DAPI (Invitrogen) and cells were analyzed through a Nikon inverted microscope attached to a laser confocal scanning system (TCS SP2, Leica Microsystems). The number of nuclei per cell at 0, 24, 48, 72 and 96 h post-synchronization was determined from 250 randomly selected trophozoites.

### Determination of nuclear DNA content

DNA content was analyzed by FACS in paraformaldehyde-fixed trophozoites after staining with propidium iodide (PI) using standard procedures. Flow cytometry was performed using the FACScalibur (Becton Dickinson) and data from 10,000 cells were recorded for each experiment and analyzed using CellQuest software (Becton Dickinson).

### Real-time microscopy

Trophozoites were synchronized as described^3^, plated on POC-R2 Chambers (LaCon, Germany) with TYI-S-33 medium supplemented with adult bovine serum (10%) for 24 h at 37 °C and visualized under a Axiovert 200 M fluorescence microscope (Zeiss Germany) (40× objective). The time-lapse images were captured with 2 s intervals, analyzed and further processed using the Axiovision v4.8 software (Zeiss, Germany).

### DNA microarrays

Total RNA was obtained from trophozoites by TRIzol (Invitrogen). cDNA synthesis and labeling with Cy3 and Cy5 fluorochromes were performed using the SuperScript™ Indirect cDNA Labeling System (Invitrogen). Then, DNA microarrays (EH-IP2008; Agilent Technologies) covering the complete *E. histolytica* genome were hybridized, washed, dried, and scanned in a GenePix 4000A apparatus as described^21^. Three biological replicates were performed and two technical replicates were hybridized. Images were analyzed using the GenePix5 software (Axon) and statistical analyses were carried out. Only genes with an expression fold change >1.5 (p ≤ 0.05) were considerate in this analysis.

### Chromatin immunoprecipitation

Assays were performed as previously described^40^ with some modifications. Briefly, trophozoites (2 × 10^7^) were resuspended in lysis buffer with protease inhibitors and sonicated to obtain 300 bp DNA fragments. Extract was clarified by centrifugation and incubated for 30 min in chromatin solubilization buffer (10 volumes) containing salmon sperm DNA, bovine serum albumin and rec-Protein G-Sepharose 4B (Invitrogene). Supernatant corresponding to soluble chromatin (0.5 ml) was incubated with anti-EhPC4 antibodies (2 μg) overnight at 4 °C and then with rec-Protein G-Sepharose 4B for 2 h at 4 °C. Subsequently, beads were sequentially washed with buffers III, IV, LiCl, and TE. The immune complexes were retrieved by incubation in elution buffer for 15 min at room temperature. To reverse cross-linking, elutes were incubated with 0.2 M NaCl for 4 h at 65 °C, followed by proteinase K and RNAse A treatment. Finally, DNA was extracted and semiquantitative RT-PCR analysis of *EhCDC48*, *EhSTG1*, *EhSMC*, *EhCWF2*, *EhODC1*, *EhNUDC*, *EhSKIP1* and *EhDUSP1* core promoters was performed using the immunoprecipitated DNA (2 μl per reaction) and specific oligonucleotides (Supplementary Table S4). All amplification reactions were performed three times by duplicate.

### Statistical analysis

Statistical analyses were performed using the GraphPad Prism version 5.0 Software package. Experimental values were reported as the mean ± standard deviation (SD) and compared with the one-way ANOVA test. Differences were considered significant when *p* < 0.05.

## Additional Information

**How to cite this article**: Cruz, O. H. *et al.* Multinucleation and Polykaryon Formation is Promoted by the EhPC4 Transcription Factor in *Entamoeba histolytica*. *Sci. Rep.* **6**, 19611; doi: 10.1038/srep19611 (2016).

## Supplementary Material

Supplementary Video 1

Supplementary Video 2

Supplementary Video 3

Supplementary Information
