# Supplementary material for: Multinucleation and Polykaryon Formation is Promoted by the EhPC4 Transcription Factor in Entamoeba histolytica
Source: Sci Rep. 2016 Jan 21;6:19611. doi: 10.1038/srep19611 (PMC4726151; doi:10.1038/srep19611)
Supplement: Supplementary Information [file srep19611-s4.pdf]

# Supplementary information

## Multinucleation and Polykaryon Formation is Promoted by EhPC4 Transcription Factor in *Entamoeba histolytica*

Olga Hernández de la Cruz[1], Laurence A. Marchat[2], Nancy Guillén[3,4], Christian Weber[3,4], Itzel López[1], José Díaz-Chávez[5], Luis Herrera[5], Arturo Rojo-Domínguez[6], Esther Orozco[7], César López-Camarillo[1]

[1]Autonomous University of Mexico City, Genomics Sciences Program, Mexico City, Mexico. [2]National Polytechnic Institute, National School of Medicine and Homeopathy, Institutional Program of Molecular Biomedicine, Biotechnology Program, Mexico City, Mexico. [3]Institut Pasteur, Cellular Biology of Parasitism Unit, Paris, France, [4]INSERM U786, Paris, France. [5]National Institute of Cancerology, Carcinogenesis Laboratory, Mexico City, Mexico. [6]Metropolitan Autonomous University, Natural Sciences Department, Mexico City, Mexico. [7]Center for Research and Advanced Studies of the National Polytechnic Institute.Department of Infectomics and Molecular Pathogenesis, Mexico City, Mexico.

***Corresponding author:** Phone: (+52) 5488-6661 ext 15307.

E-mail address: cesar.lopez@uacm.edu.mx (Dr. César López-Camarillo).

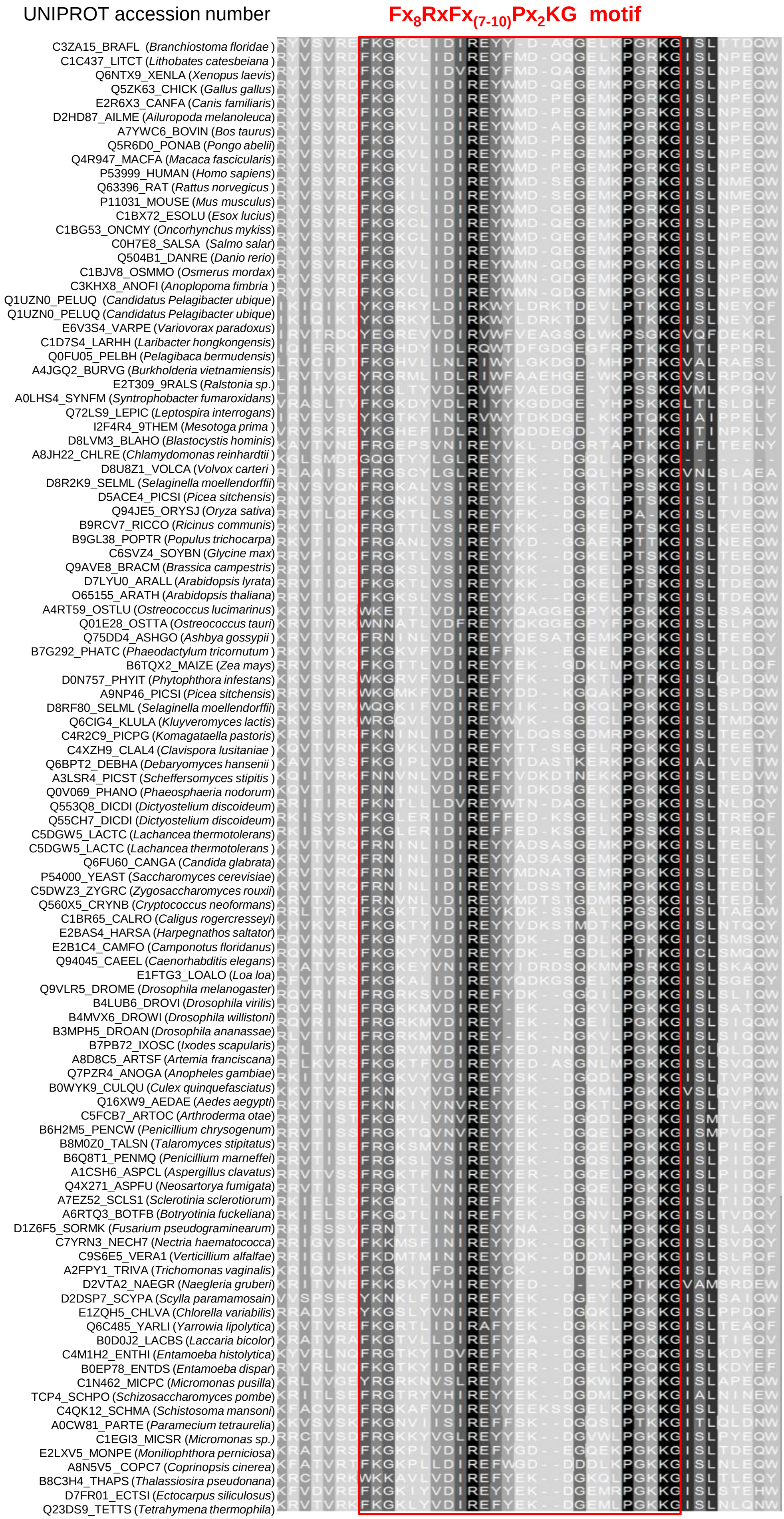


**Supplementary figure 1. Multiple sequence alignment of EhPC4 and orthologous proteins.** White letters on black background represent identical residues in the sequences aligned. White letters on a gray background are the similar residues. The $Fx_8RxFx_{(7-10)}Px_2KG$ motif is depicted in red box. Alignment image created using the multiple alignment software ClustalW2 and BioEdit Sequence Alignment Editor.

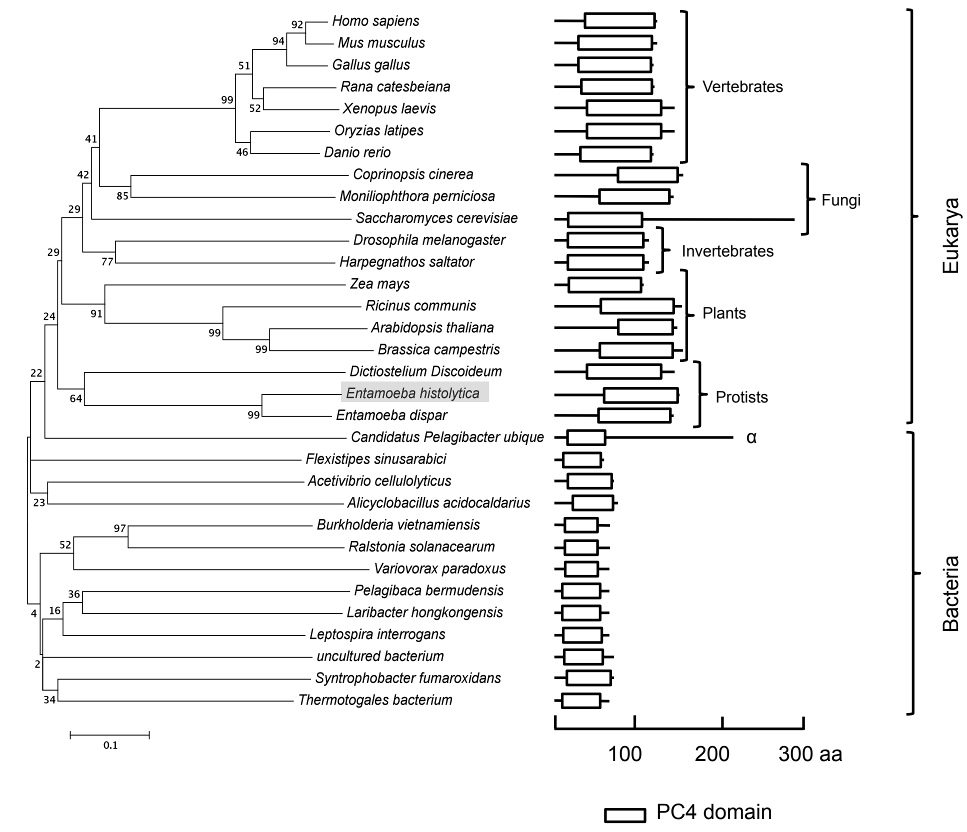


**Supplementary figure S2.** Phylogenetic relationships among PC4 proteins from diverse organisms. The evolutionary history was inferred using the Neighbor-Joining method and 1000 replicates in order to obtain the bootstrap consensus tree. Numbers at the nodes indicate the statistical support of the branching order by the bootstrap criterion. The bar at the bottom of phylogram is a reference to branch length, which is proportional to the amount of inferred evolutionary change. Right, molecular organization of PC4 related proteins. Open box, predicted PC4 domain.

A

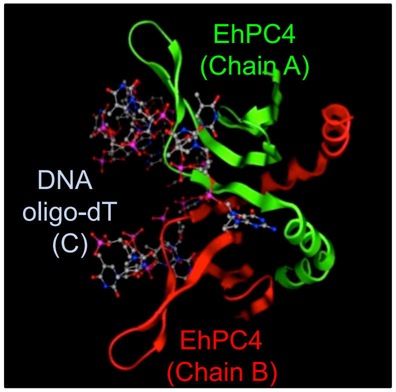


B

| Residue chain A Wild type | Energy (mutation to alanine) Kcal/mol | Energy difference (Wt -mutant) Kcal/mol | Residue Chain B Wild type | Energy (mutation to alanine) Kcal/mol | Energy difference (Wt-mutant) Kcal/mol | Chain A+ Chain B Kcal/mol |
|---|---|---|---|---|---|---|
| K97 | A97= -392.726 | 15.536 | K97 | A197= -409.374 | -1.112 | 14.424 |
| F104 | A104= -403.640 | 4.622 | F104 | A104= -402.694 | 5.568 | 10.190 |
| R105 | A105= -388.840 | 19.422 | R105 | A105= -408.318 | -0.056 | 19.366 |
| R113 | A113= -356.117 | 52.145 | R113 | A113= -386.439 | 21.823 | 73.968 |
| Y116 | A116= -420.697 | -12.435 | Y116 | A116= -405.032 | 3.230 | -9.205 |
| R118 | A118= 419.504 | -11.242 | R118 | A118= -350.034 | 58.228 | 46.986 |
| K123 | A123= -391.658 | 16.604 | K123 | A123= -401.070 | 7.129 | 23.796 |
| Q126 | A126= -391.669 | 16.593 | Q126 | A126= -400.703 | 7.559 | 24.152 |
| **K127** | **A127= -350.896** | **57.366** | **K127** | **A127=-330.995** | **77.267** | **134.633** |
| I129 | A129= -406.930 | 1.332 | I129 | A129= -408.471 | -0.209 | 1.123 |

Energy interaction (wild type= -408.262)

**Supplementary figure S3. Effect of the substitution of independent residues for alanine on the interaction energy**. (A) Molecular modeling of CTD-EhPC4 dimer in complex with an oligonucleotide 19-mer. (B) Effect of substitutions for alanine in the interaction energy.

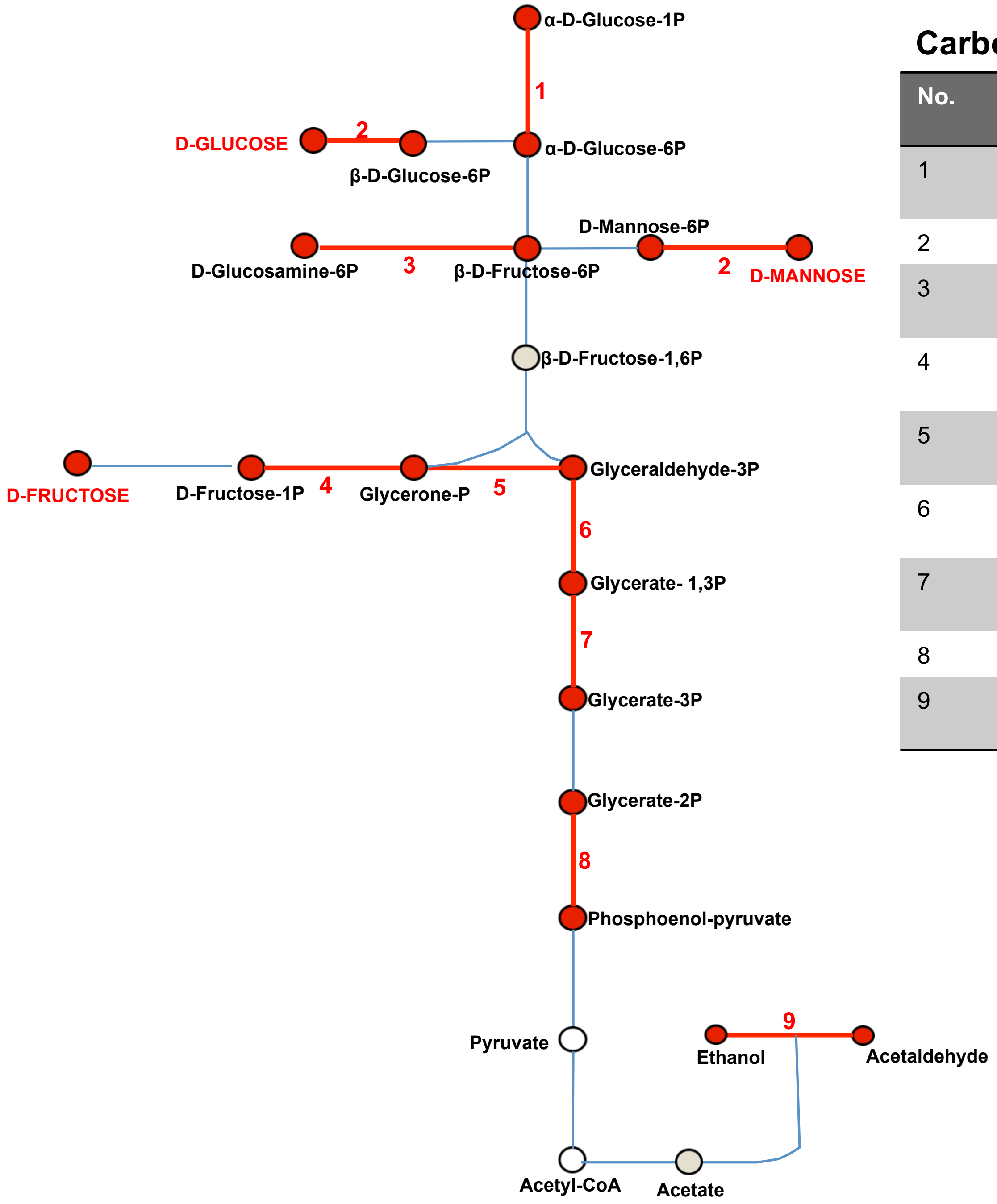


## Carbohydrate metabolism

| No. | Enzyme | Gene ID | Fold change |
|---|---|---|---|
| 1 | Phosphoglucomutase (PGM) | EHI_110120 | 1.62 |
| 2 | Hexokinase (HK) | EHI_098290 | 1.5 |
| 3 | Glucosamine-6-phosphate isomerase (GPI) | EHI_174640 | 1.86 |
| 4 | Fructose 1,6 bisphosphate aldolase (ALDO) | EHI_098570 | 2.04 |
| 5 | Triosephosphate isomerase (TPI) | EHI_056480 | 2.04 |
| 6 | Glyceraldehyde 3 phosphate dehydrogenase (GAP) | EHI_187020<br>EHI_008200 | 3.04<br>2.9 |
| 7 | Phosphoglycerate kinase (PGK) | EHI_188180 | 2.45 |
| 8 | Enolase (ENO) | EHI_130700 | 2.3 |
| 9 | NADP-dependent alcohol dehydrogenase (ADH) | EHI_023110 | 2.01 |

**Supplementary figure S4**. Overexpression of EhNUDC stimulates carbohydrate metabolism in *E. histolytica.* Reactions of glucose, fructose and mannose utilization that are catalyzed by the up-regulated genes in EhPC4-overexpressing trophozoites are indicated.

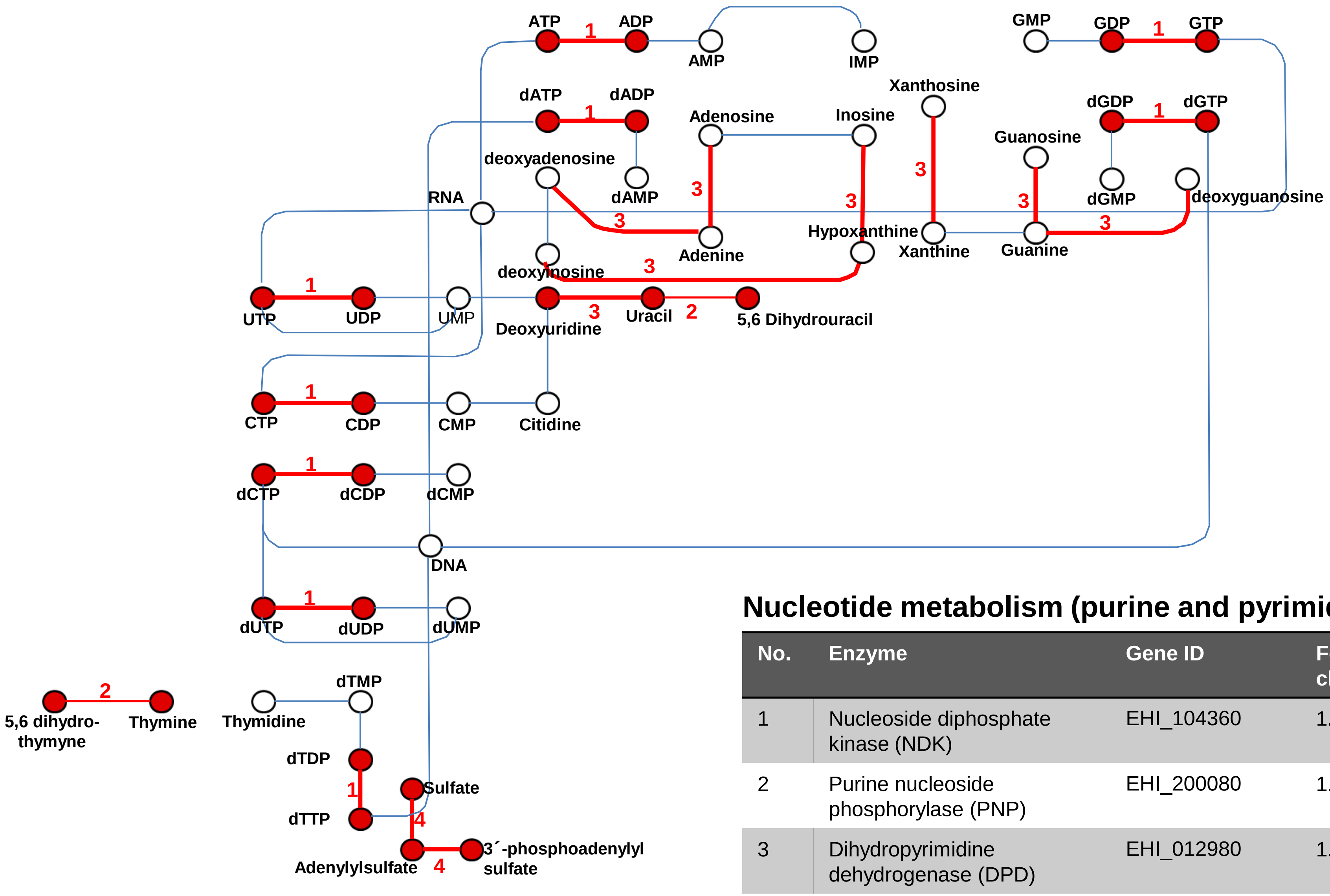


## Nucleotide metabolism (purine and pyrimidine)

| No. | Enzyme | Gene ID | Fold change |
|---|---|---|---|
| 1 | Nucleoside diphosphate kinase (NDK) | EHI_104360 | 1.72 |
| 2 | Purine nucleoside phosphorylase (PNP) | EHI_200080 | 1.59 |
| 3 | Dihydropyrimidine dehydrogenase (DPD) | EHI_012980 | 1.95 |
| 4 | Sufate adenylyltransferase (PAPSS2) | EHI_197160 | 3.17 |

**Supplementary figure S5.** Overexpression of EhNUDC stimulates nucleotide (purine and pyrimidine) metabolism in *E. histolytica*. Reactions that are catalyzed by the up-regulated genes in EhPC4-overexpressing trophozoites are indicated.

**Supplementary Table S1.** Up-regulated genes in Myc-EhPC4 cells.

| Description | Gene.ID | FC | BY | rawp |
|---|---|---|---|---|
| Heat shock protein, Hsp20 family | EHI_055680 | 8.76773 | 1.06E-04 | 4.43E-07 |
| Heat shock protein 101 | EHI_178230 | 7.248758 | 1.13E-04 | 4.81E-07 |
| Heat shock protein, putative | EHI_156560 | 6.937912 | 5.16E-05 | 1.58E-07 |
| Heat shock protein 101 | EHI_076480 | 6.906415 | 9.61E-05 | 3.85E-07 |
| EhCP A4 | EHI_050570 | 6.824281 | 2.51E-05 | 5.58E-08 |
| Heat shock protein 101 | EHI_094470 | 6.655094 | 6.51E-05 | 2.27E-07 |
| AIG1 family protein | EHI_180390 | 6.050634 | 1.38E-05 | 1.98E-08 |
| Chaperone clpB | EHI_155060 | 5.935963 | 1.32E-04 | 5.84E-07 |
| Heat shock protein70, hsp70A2 | EHI_192440 | 5.685829 | 1.87E-04 | 9.01E-07 |
| Chaperone clpB | EHI_094680 | 5.453589 | 7.17E-05 | 2.57E-07 |
| Heat shock protein 101 | EHI_183680 | 5.300941 | 7.02E-05 | 2.49E-07 |
| Chaperone clpB | EHI_090840 | 5.245448 | 2.00E-05 | 3.80E-08 |
| Calcium binding protein | 4.m00695 | 5.090119 | 9.29E-06 | 1.01E-08 |
| Calcium-binding protein 2 | EHI_010020 | 4.911769 | 8.40E-06 | 7.32E-09 |
| DnaJ family protein | EHI_187000 | 4.68556 | 7.20E-05 | 2.59E-07 |
| Heat shock protein 70 | EHI_026590 | 4.640024 | 1.52E-05 | 2.33E-08 |
| Heat shock protein70, hsp70A2 | EHI_061640 | 4.564673 | 3.85E-05 | 1.05E-07 |
| 20 kDa antigen related protein | EHI_056490 | 4.293106 | 1.71E-05 | 2.71E-08 |
| Cysteine synthase A (cysK) | EHI_060340 | 4.193991 | 1.87E-05 | 3.36E-08 |
| EhCP A6 | EHI_151440 | 4.10936 | 1.36E-05 | 1.89E-08 |
| Heat shock protein 101 | EHI_063440 | 4.090962 | 9.71E-06 | 1.11E-08 |
| Heat shock protein 90 | EHI_196940 | 4.083139 | 5.27E-06 | 2.63E-09 |
| Heat shock protein70, hsp70A2 | EHI_132530 | 4.071865 | 2.18E-05 | 4.43E-08 |
| Ubiquitin | EHI_083410 | 3.910963 | 2.19E-06 | 4.32E-10 |
| Helix-turn-helix, EDF1 | EHI_093330 | 3.866882 | 2.31E-05 | 4.83E-08 |
| Heat shock protein 101 | EHI_013550 | 3.513846 | 4.96E-05 | 1.49E-07 |
| Translation initiation factor eIF 5A (eif5A) | EHI_177460 | 3.487082 | 1.22E-05 | 1.54E-08 |
| Sgt1 | EHI_117820 | 3.445039 | 3.07E-05 | 7.64E-08 |
| Sulfotransferase | EHI_197340 | 3.371605 | 1.87E-05 | 3.40E-08 |
| Sulfate adenylyltransferase | EHI_197160 | 3.178127 | 5.94E-06 | 4.06E-09 |
| Heat shock protein70, hsp70A2 | EHI_108130 | 3.171272 | 3.21E-05 | 8.31E-08 |
| DnaJ family protein | EHI_151260 | 3.152776 | 4.14E-05 | 1.19E-07 |
| Heat shock protein70, hsp70A2 | EHI_132540 | 3.14286 | 9.68E-05 | 3.89E-07 |
| Myb like DNA binding domain containing | EHI_129790 | 3.119383 | 6.88E-06 | 5.34E-09 |
| Hsp90-like protein | EHI_136350 | 3.07375 | 5.60E-06 | 3.22E-09 |
| Rab family GTPase | EHI_164900 | 3.056108 | 4.00E-05 | 1.12E-07 |
| Glyceraldehyde 3 phosphate dehydrogenase | EHI_187020 | 3.041685 | 1.25E-06 | 1.65E-11 |
| Dual specificity protein phosphatase | EHI_175490 | 3.039278 | 2.11E-06 | 2.84E-10 |
| Iron sulfur flavoprotein | EHI_022600 | 3.031907 | 2.11E-06 | 2.39E-10 |
| Glyceraldehyde 3 phosphate dehydrogenase | EHI_008200 | 2.986676 | 2.11E-06 | 1.61E-10 |
| Eukaryotic translation initiation factor 6 | EHI_006170 | 2.981978 | 2.11E-06 | 3.51E-10 |

| | | | | |
|---|---|---|---|---|
| SMC-domain containig protein | EHI_187190 | 2.975095 | 5.86E-05 | 1.89E-07 |
| Cyst wall specific glyco protein Jacob | EHI_136360 | 2.935446 | 3.52E-05 | 9.38E-08 |
| Heat shock protein70, hsp70A2 | EHI_015390 | 2.92836 | 2.64E-05 | 6.22E-08 |
| DadA family oxidoreductase | EHI_028940 | 2.895756 | 4.35E-06 | 1.72E-09 |
| BAR/SH3 domain containing protein | EHI_035790 | 2.877904 | 1.06E-05 | 1.27E-08 |
| Iron sulfur flavoprotein | EHI_067720 | 2.852429 | 2.74E-06 | 6.12E-10 |
| Rab family GTPase | EHI_180410 | 2.838496 | 2.41E-04 | 1.28E-06 |
| Malate dehydrogenase | EHI_092450 | 2.824192 | 2.47E-05 | 5.44E-08 |
| Cyst wall specific glycoprotein Jacob | EHI_028930 | 2.816447 | 4.13E-05 | 1.18E-07 |
| SGS domain protein | EHI_050290 | 2.816141 | 5.60E-06 | 3.55E-09 |
| Ubiquitin conjugating enzyme family | EHI_048700 | 2.749335 | 2.91E-05 | 7.12E-08 |
| NudC | EHI_023890 | 2.736238 | 6.51E-05 | 2.26E-07 |
| Fes1-domain containing protein | EHI_061760 | 2.649043 | 3.01E-06 | 7.13E-10 |
| NAD-dependent epímerase/Dehydratase | EHI_017660 | 2.643872 | 1.64E-06 | 4.32E-11 |
| Ubiquitin conjugating enzyme family | EHI_160740 | 2.587297 | 8.89E-06 | 8.77E-09 |
| Heat shock protein, Hsp20 family | EHI_125830 | 2.563051 | 2.34E-05 | 4.96E-08 |
| Hsp70 | EHI_192510 | 2.536149 | 5.37E-05 | 1.67E-07 |
| Proline synthetase associated protein | EHI_188790 | 2.523961 | 1.36E-05 | 1.90E-08 |
| Ubiquitin | EHI_178340 | 2.506538 | 5.55E-06 | 2.92E-09 |
| Copine 9 | EHI_137110 | 2.498739 | 2.11E-06 | 3.15E-10 |
| Actin binding protein, cofilin/tropomyosin family | EHI_186840 | 2.488925 | 8.40E-06 | 7.08E-09 |
| Ras family GTPase | EHI_093760 | 2.480369 | 2.11E-06 | 3.75E-10 |
| Ubiquitin conjugating enzyme family | EHI_070750 | 2.480125 | 4.03E-06 | 1.27E-09 |
| Iron-sulfur flavoprotein | EHI_022270 | 2.470803 | 6.20E-05 | 2.09E-07 |
| Ubiquitin like, | EHI_103510 | 2.451989 | 3.72E-06 | 1.12E-09 |
| Phosphoglycerate kinase (pgk) | EHI_188180 | 2.451013 | 6.31E-06 | 4.60E-09 |
| Ornithine decarboxylase | EHI_100430 | 2.421524 | 4.35E-06 | 1.78E-09 |
| Hypothetical protein | EHI_009900 | 2.375187 | 8.40E-06 | 7.50E-09 |
| Carbonic anhydrase | EHI_073380 | 2.37082 | 5.60E-06 | 3.28E-09 |
| Thioredoxin (TRX) | EHI_004490 | 2.36349 | 1.32E-05 | 1.73E-08 |
| Hydroxylamine reductase | EHI_004600 | 2.335483 | 2.39E-05 | 5.18E-08 |
| Gal/GalNAc lectin Igl2 | EHI_183000 | 2.330014 | 5.30E-06 | 2.72E-09 |
| Iron sulfur flavoprotein | EHI_103260 | 2.320236 | 4.53E-06 | 2.08E-09 |
| Enolase | EHI_130700 | 2.316064 | 2.56E-05 | 5.75E-08 |
| Chemoreceptor glutamine deamidase CheD | EHI_192430 | 2.30639 | 4.35E-06 | 1.94E-09 |
| MSF transporter | EHI_009500 | 2.296668 | 2.43E-06 | 5.10E-10 |
| Sulfotransferase | EHI_031640 | 2.295526 | 9.89E-05 | 4.02E-07 |
| Heat shock protein, Hsp20 family | EHI_193390 | 2.285371 | 1.17E-05 | 1.46E-08 |
| RNA 3' terminal phosphate cyclase | EHI_177570 | 2.266218 | 2.35E-05 | 5.08E-08 |
| Malic enzyme | EHI_050330 | 2.232511 | 1.94E-06 | 8.56E-11 |
| Heat shock protein 70 | EHI_052860 | 2.230587 | 3.21E-05 | 8.37E-08 |
| EhPC4 | EHI_192520 | 2.22746 | 1.10E-04 | 4.68E-07 |
| Helicase SWR1 | EHI_124540 | 2.212942 | 2.86E-05 | 6.93E-08 |
| Pyrophosphate dependent phosphofructokinase | EHI_000730 | 2.203685 | 4.35E-06 | 1.81E-09 |
| Cell surface protein | EHI_114770 | 2.199277 | 5.84E-05 | 1.86E-07 |

| | | | | |
|---|---|---|---|---|
| Lipopolysaccharide-induced transcription factor regulating TNF-alpha | EHI_003260 | 2.182988 | 2.17E-04 | 1.12E-06 |
| High mobility group protein | EHI_098750 | 2.151992 | 6.20E-05 | 2.08E-07 |
| Calcium-binding protein 1 | EHI_120900 | 2.149705 | 5.60E-06 | 3.63E-09 |
| Hypothetical protein | EHI_010260 | 2.126326 | 5.60E-06 | 3.32E-09 |
| Periplasmic beta-glucosidase | EHI_066750 | 2.126204 | 2.35E-05 | 5.07E-08 |
| Aspartate ammonia lyase | EHI_150390 | 2.115875 | 8.40E-06 | 7.50E-09 |
| EMB2261 | EHI_023150 | 2.113032 | 6.31E-06 | 4.53E-09 |
| Surface antigen ariel1 | EHI_080200 | 2.111899 | 1.39E-04 | 6.24E-07 |
| GRIP domain-containing protein, RUD3 | EHI_169670 | 2.088252 | 1.78E-04 | 8.48E-07 |
| Aspartyl tRNA synthetase (aspS) | EHI_175050 | 2.088124 | 4.08E-06 | 1.38E-09 |
| Sulfotransferase | EHI_166030 | 2.07592 | 1.79E-05 | 3.11E-08 |
| Hypothetical protein | EHI_135600 | 2.075443 | 2.17E-04 | 1.12E-06 |
| AMMECR1 family | EHI_143570 | 2.053269 | 1.06E-04 | 4.45E-07 |
| Acetyl CoA synthetase | EHI_178960 | 2.051847 | 3.06E-06 | 7.64E-10 |
| Fructose 1,6 bisphosphate aldolase | EHI_098570 | 2.046977 | 9.45E-05 | 3.74E-07 |
| Triosephosphate isomerase | EHI_056480 | 2.045719 | 4.35E-06 | 1.93E-09 |
| Surface antigen BspA-like | EHI_056350 | 2.040988 | 1.52E-05 | 2.33E-08 |
| Hypothetical protein | EHI_163490 | 2.016749 | 5.11E-06 | 2.48E-09 |
| Leucine rich repeat protein, BspA family | EHI_024580 | 2.013854 | 5.05E-06 | 2.39E-09 |
| NADP dependent alcohol dehydrogenase | EHI_023110 | 2.010902 | 1.86E-05 | 3.31E-08 |
| Alcohol dehydrogenase | EHI_125950 | 1.995815 | 3.25E-06 | 8.55E-10 |
| Aspartate ammonia lyase | EHI_082270 | 1.989946 | 8.69E-06 | 8.22E-09 |
| Copine 8 | EHI_096790 | 1.984381 | 1.51E-05 | 2.29E-08 |
| Ribosomal protein S30 | EHI_023400 | 1.970073 | 2.78E-05 | 6.64E-08 |
| Dihydropyrimidine dehydrogenase | EHI_012980 | 1.958907 | 9.87E-06 | 1.15E-08 |
| Synaptic glycoprotein SC2 | EHI_135110 | 1.939545 | 7.02E-05 | 2.49E-07 |
| Sulfotransferase | EHI_140740 | 1.939407 | 2.69E-05 | 6.40E-08 |
| Hsc70 interacting protein | EHI_158050 | 1.929658 | 1.54E-05 | 2.41E-08 |
| Asparaginyl tRNA synthetase (asnS) | EHI_126920 | 1.915217 | 2.21E-04 | 1.14E-06 |
| EhCP1 | EHI_074180 | 1.914549 | 1.04E-04 | 4.28E-07 |
| Surface antigen ariel1 | EHI_131360 | 1.904506 | 3.06E-05 | 7.57E-08 |
| Calmodulin | EHI_100270 | 1.899032 | 2.98E-05 | 7.33E-08 |
| Filamin-binding LIM protein 1 | EHI_069060 | 1.890248 | 1.38E-03 | 1.28E-05 |
| 70 kDa peptidyl prolyl isomerase | EHI_178850 | 1.874912 | 2.60E-05 | 5.92E-08 |
| Surface antigen ariel1 | EHI_172850 | 1.869849 | 7.62E-05 | 2.82E-07 |
| Glucosamine 6 phosphate isomerase | EHI_174640 | 1.868506 | 1.74E-05 | 2.90E-08 |
| Ras family GTPase | EHI_154240 | 1.850227 | 1.79E-05 | 3.09E-08 |
| Cysteine desulfurase | EHI_136380 | 1.847157 | 1.96E-04 | 9.68E-07 |
| Ubiquitin conjugating enzyme family | EHI_083560 | 1.844057 | 1.71E-05 | 2.75E-08 |
| Dcn1p | EHI_152590 | 1.841838 | 1.93E-04 | 9.48E-07 |
| Skp1A | EHI_134960 | 1.840115 | 6.88E-06 | 5.25E-09 |
| PH domain containing protein | EHI_117920 | 1.835931 | 2.60E-04 | 1.44E-06 |
| E3 ubiquitin protein ligase BRE1 | EHI_074070 | 1.814448 | 3.14E-05 | 8.02E-08 |
| Actophorin | EHI_197480 | 1.806128 | 3.72E-06 | 1.12E-09 |
| Ubiquitin | EHI_166800 | 1.8061 | 1.37E-05 | 1.92E-08 |
| Proteasome alpha subunit | EHI_163650 | 1.801178 | 1.36E-05 | 1.89E-08 |

| | | | | |
|---|---|---|---|---|
| tRNA-splicing ligase RtcB homolog 2 | EHI_184560 | 1.788487 | 3.77E-05 | 1.02E-07 |
| TFIID subunit | EHI_137090 | 1.784791 | 1.09E-05 | 1.33E-08 |
| Leucine rich repeat protein, BspA family | EHI_082060 | 1.781716 | 2.20E-05 | 4.55E-08 |
| Proteasome alpha subunit | EHI_090000 | 1.778393 | 6.30E-06 | 4.39E-09 |
| Glutamyl tRNA synthetase | EHI_155570 | 1.756698 | 1.51E-04 | 6.92E-07 |
| Rab family GTPase | EHI_114210 | 1.753429 | 4.34E-05 | 1.25E-07 |
| Methionine gamma lyase | EHI_144610 | 1.751452 | 5.39E-04 | 3.73E-06 |
| F actin capping protein subunit alpha | EHI_140640 | 1.749724 | 1.40E-03 | 1.30E-05 |
| EF hand calcium binding domain containing protein | EHI_197510 | 1.749023 | 1.04E-05 | 1.23E-08 |
| Chaperone protein dnaK | EHI_037230 | 1.742242 | 2.41E-04 | 1.28E-06 |
| Grainin 2 | EHI_111720 | 1.73186 | 8.96E-06 | 8.95E-09 |
| Nucleoside diphosphate kinase | EHI_104360 | 1.72778 | 1.99E-05 | 3.76E-08 |
| Methionine gamma lyase | EHI_057550 | 1.723721 | 9.05E-05 | 3.55E-07 |
| Proteolipid membrane potential modulator | EHI_022900 | 1.722794 | 2.55E-05 | 5.69E-08 |
| Rho family GTPase | EHI_129750 | 1.721769 | 3.21E-05 | 8.35E-08 |
| Cysteine rich surface protein | EHI_197360 | 1.719703 | 3.21E-05 | 8.31E-08 |
| Glucosamine 6 phosphate N acetyltransferase | EHI_080280 | 1.709959 | 4.27E-04 | 2.73E-06 |
| Galactose specific adhesin light subunit | EHI_049690 | 1.706383 | 8.40E-06 | 7.18E-09 |
| Ras family GTPase | EHI_137700 | 1.701221 | 8.66E-05 | 3.32E-07 |
| Obg-like ATPase 1 | EHI_148270 | 1.699307 | 1.38E-05 | 2.04E-08 |
| Actin | EHI_107290 | 1.698642 | 7.38E-04 | 5.73E-06 |
| Actin | EHI_159150 | 1.695353 | 5.85E-05 | 1.87E-07 |
| Actin | EHI_182900 | 1.684555 | 1.71E-05 | 2.70E-08 |
| Pyruvate phosphate dikinase (ppdK) | EHI_009530 | 1.68369 | 1.86E-05 | 3.30E-08 |
| Actinin like protein | EHI_164440 | 1.682636 | 3.07E-04 | 1.80E-06 |
| Ubiquitin fusion degradation protein 1 | EHI_125920 | 1.680541 | 1.39E-04 | 6.25E-07 |
| Ubiquitin conjugating enzyme E2 6 | EHI_082280 | 1.668011 | 6.47E-05 | 2.23E-07 |
| Metal dependent phosphohydrolase | EHI_195060 | 1.667516 | 3.37E-04 | 2.03E-06 |
| Histone H4 | EHI_023230 | 1.659423 | 2.83E-03 | 3.21E-05 |
| Zinc finger protein 622 | EHI_154230 | 1.656656 | 4.87E-04 | 3.28E-06 |
| RAD23 protein | EHI_001400 | 1.654035 | 3.55E-04 | 2.16E-06 |
| Cell division cycle protein 48 | EHI_045120 | 1.653682 | 9.29E-06 | 9.95E-09 |
| (2r) phospho 3 sulfolactate synthase | EHI_026480 | 1.649916 | 1.17E-05 | 1.46E-08 |
| ARIEL1 | 160.m00087 | 1.649043 | 4.04E-04 | 2.56E-06 |
| Proteasome beta subunit | EHI_011870 | 1.646393 | 7.10E-04 | 5.44E-06 |
| Cullin | EHI_118180 | 1.643174 | 7.60E-05 | 2.80E-07 |
| Actin | EHI_163750 | 1.640469 | 1.14E-03 | 9.97E-06 |
| Actin 2 protein | EHI_161200 | 1.635811 | 4.59E-05 | 1.36E-07 |
| Phosphoglucomutase | EHI_110120 | 1.628724 | 4.08E-05 | 1.15E-07 |
| TBP2 | EHI_112050 | 1.61979 | 6.13E-04 | 4.41E-06 |
| EhMP24 1 methionine aminopeptidase | EHI_126880 | 1.616879 | 1.73E-04 | 8.23E-07 |
| Memo-like protein | EHI_024510 | 1.6127546 | 2.20E-05 | 4.56E-08 |
| Calcium dependent protein kinase 2 | EHI_137120 | 1.610936 | 1.03E-03 | 8.85E-06 |
| Pyruvate:ferredoxin oxidoreductase | EHI_051060 | 1.608403 | 3.55E-04 | 2.17E-06 |
| | EHI_050270 | 1.607109 | 2.65E-05 | 6.28E-08 |

| | | | | |
|---|---|---|---|---|
| Ribosomal protein S30 | EHI_088600 | 1.604312 | 6.20E-05 | 2.10E-07 |
| COP-9 SigNalosome subunit family member (csn-5) | EHI_050500 | 1.599101 | 4.19E-04 | 2.66E-06 |
| High mobility group protein B3 | EHI_087410 | 1.599027 | 2.54E-04 | 1.39E-06 |
| Purine nucleoside phosphorylase | EHI_200080 | 1.596369 | 2.64E-05 | 6.20E-08 |
| Rap GTPase-activating protein | EHI_137690 | 1.594249 | 9.67E-04 | 8.13E-06 |
| Translation initiation factor eIF 5A (eif5A) | EHI_186480 | 1.593164 | 1.52E-05 | 2.36E-08 |
| Peptidyl prolyl cis trans isomerase | EHI_125840 | 1.593091 | 9.75E-03 | 1.50E-04 |
| 3 oxoacyl (acyl carrier protein) reductase | EHI_186920 | 1.587006 | 1.73E-04 | 8.21E-07 |
| Enhancer binding protein 1 | EHI_121780 | 1.584608 | 2.11E-05 | 4.27E-08 |
| Proteasome subunit beta Type 4 precursor | EHI_137970 | 1.583067 | 2.58E-04 | 1.43E-06 |
| SRPK1 | EHI_035500 | 1.57812 | 2.06E-05 | 4.08E-08 |
| High mobility group protein | EHI_086110 | 1.570097 | 9.01E-03 | 1.36E-04 |
| SCAI | EHI_186790 | 1.566906 | 6.47E-05 | 2.22E-07 |
| Diacylglycerol kinase | EHI_117800 | 1.556544 | 6.97E-04 | 5.29E-06 |
| Molybdenum cofactor synthesis protein3 | EHI_118040 | 1.548053 | 4.00E-05 | 1.13E-07 |
| Endoribonuclease L_PSP | EHI_087570 | 1.546821 | 5.16E-05 | 1.58E-07 |
| Hexokinase | EHI_098290 | 1.542745 | 6.42E-04 | 4.66E-06 |
| Poly (A) binding protein | EHI_177560 | 1.537549 | 2.00E-05 | 3.84E-08 |
| 26S protease regulatory subunit 7 | EHI_080890 | 1.534371 | 2.88E-04 | 1.65E-06 |
| Rho GTPase activating protein | EHI_187110 | 1.534321 | 1.07E-04 | 4.53E-07 |
| Fe S cluster assembly protein NifU | EHI_049620 | 1.529576 | 3.73E-05 | 1.00E-07 |
| Seryl tRNA synthetase | EHI_000690 | 1.525414 | 6.52E-04 | 4.76E-06 |
| 40S ribosomal protein S13 | EHI_112880 | 1.509673 | 4.21E-02 | 9.28E-04 |
| TPR repeat protein, STIP1 | EHI_023300 | 1.509121 | 2.14E-04 | 1.09E-06 |
| 60S acidic ribosomal protein P2 | EHI_186830 | 1.504835 | 2.79E-05 | 6.70E-08 |
| Inorganic pyrophosphatase | EHI_124880 | 1.504483 | 1.35E-04 | 6.02E-07 |
| Cell cycle control protein cwf12 | EHI_187260 | 1.503032 | 8.41E-05 | 3.21E-07 |

**Supplementary Table S2.** Down-regulated genes in Myc-*Eh*PC4 cells.

| Description | Gene.ID | FC | BY | Rawp |
|---|---|---|---|---|
| N system aminoacid transporter 1 | EHI_050900 | -9.43 | 2.62E-05 | 6.06E-08 |
| Nonpathogenic pore forming peptide precursor | EHI_169350 | -3.98 | 4.00E-04 | 2.52E-06 |
| Probably a phosphatidate cytidylytransferase family | EHI_163240 | -3.17 | 2.11E-06 | 3.06E-10 |
| D 3 phosphoglycerate dehydrogenase | EHI_060860 | -2.85 | 1.95E-05 | 3.60E-08 |
| S adenosylmethionine syntase | EHI_004920 | -2.72 | 6.93E-06 | 5.56E-09 |
| Hypotetical protein | EHI_143070 | -2.66 | 8.69E-06 | 8.17E-09 |
| S adenosylmethionine syntase | EHI_174250 | -2.65 | 7.38E-04 | 5.72E-06 |
| Cysteine proteinase 2 precursor | EHI_132650 | -2.62 | 2.11E-06 | 3.32E-10 |
| Ganglioside gm2 activator protein | EHI_151800 | -2.54 | 4.38E-04 | 2.85E-06 |
| Ribonuclease putative | EHI_156310 | -2.52 | 2.11E-06 | 3.09E-10 |
| D phosphoglycerate dehydrogenase | EHI_116830 | -2.44 | 1.95E-05 | 3.62E-08 |
| Integral membrane protein | EHI_131130 | -2.39 | 5.80E-06 | 3.89E-09 |
| Topoisomerase | EHI_048140 | -2.32 | 3.42E-06 | 9.44E-10 |
| EhCP A7 cysteine proteinase | EHI_039610 | -2.32 | 8.73E-06 | 8.41E-09 |
| Protein kinase similar to intersectin 2 isoform 3 | EHI_012020 | -2.31 | 6.31E-06 | 4.64E-09 |
| Hypotetical protein | EHI_079280 | -2.3 | 8.28E-06 | 6.75E-09 |
| Cysteine proteinase, putative | EHI_144040 | -2.29 | 4.08E-06 | 1.40E-09 |
| DEAD/DEAH box helicase | EHI_036900 | -2.28 | 8.65E-06 | 7.85E-09 |
| Lysozyme | EHI_096570 | -2.27 | 5.92E-05 | 1.93E-07 |
| Methionine gamma lyase | EHI_142250 | -2.26 | 8.73E-06 | 8.49E-09 |
| Cysteine proteinase, putative | EHI_144050 | -2.21 | 5.60E-06 | 3.59E-09 |
| Hypotetical protein | EHI_093740 | -2.14 | 1.94E-06 | 1.02E-10 |
| Long chain fatty acid CoA ligase | EHI_079300 | -2.13 | 5.60E-06 | 3.68E-09 |
| Ribonuclease | EHI_169300 | -2.12 | 1.27E-04 | 5.54E-07 |
| Cysteine proteinase, putative | EHI_010850 | -2.1 | 4.27E-06 | 1.57E-09 |
| Merozoite surface protein-1 | EHI_053850 | -2.06 | 1.71E-05 | 2.77E-08 |
| Fe hydrogenase | EHI_005060 | -2.05 | 1.71E-05 | 2.79E-08 |
| M protein, serotype 5 precursor | EHI_188170 | -2.03 | 6.88E-06 | 5.32E-09 |
| Rab family GTPase | EHI_127380 | -2.02 | 3.48E-05 | 9.18E-08 |
| Hypothetical protein | EHI_058110 | -1.99 | 5.60E-06 | 3.19E-09 |
| Polyadenylate binding protein, putative | EHI_033250 | -1.96 | 2.03E-05 | 3.97E-08 |
| Rho family GTPase | EHI_192450 | -1.94 | 2.11E-06 | 3.87E-10 |
| S adenosylmethionine synthetase, putative | EHI_195110 | -1.94 | 6.20E-05 | 2.11E-07 |
| Hypothetical protein | EHI_136410 | -1.93 | 4.08E-06 | 1.45E-09 |
| Hypothetical protein, conserved | EHI_191770 | -1.92 | 1.46E-05 | 2.17E-08 |
| Hypothetical protein | EHI_096360 | -1.91 | 5.60E-06 | 3.44E-09 |
| Hypothetical protein | EHI_151390 | -1.91 | 1.86E-05 | 3.32E-08 |
| Hypothetical protein | EHI_194550 | -1.9 | 9.29E-06 | 9.83E-09 |
| Hypothetical protein | EHI_020080 | -1.89 | 1.07E-05 | 1.30E-08 |
| Hypothetical protein, conserved | EHI_030790 | -1.87 | 8.03E-05 | 3.00E-07 |
| RNA binding protein, putative | EHI_196590 | -1.87 | 1.76E-05 | 2.95E-08 |
| choline/ethanolamine kinase, putative | EHI_148580 | -1.86 | 1.30E-05 | 1.67E-08 |

| | | | | |
|---|---|---|---|---|
| Fatty acid elongase, putative | EHI_111000 | -1.86 | 2.61E-05 | 5.97E-08 |
| Hypothetical protein, conserved | EHI_095850 | -1.86 | 8.69E-06 | 8.14E-09 |
| Hypothetical protein | EHI_097960 | -1.84 | 3.96E-05 | 1.10E-07 |
| Hypothetical protein, conserved | EHI_092620 | -1.84 | 4.83E-05 | 1.43E-07 |
| Hypothetical protein | EHI_125150 | -1.82 | 8.40E-06 | 7.38E-09 |
| Hypothetical protein | EHI_166360 | -1.82 | 2.47E-05 | 5.43E-08 |
| Hypothetical protein | EHI_155550 | -1.81 | 1.78E-05 | 3.03E-08 |
| NAD(P) transhydrogenase subunit alpha, putative | EHI_014030 | -1.8 | 4.35E-06 | 1.94E-09 |
| V type ATPase, C subunit, putative | EHI_059840 | -1.8 | 2.23E-04 | 1.15E-06 |
| Hypothetical protein | EHI_037160 | -1.79 | 6.20E-05 | 2.10E-07 |
| Hypothetical protein | EHI_181570 | -1.78 | 9.60E-06 | 1.07E-08 |
| Hypothetical protein | EHI_021190 | -1.78 | 1.38E-05 | 1.98E-08 |
| Hypothetical protein, conserved | EHI_160010 | -1.77 | 1.49E-05 | 2.23E-08 |
| Hypothetical protein | EHI_096340 | -1.76 | 1.38E-05 | 2.01E-08 |
| Hypothetical protein | EHI_160980 | -1.76 | 5.09E-05 | 1.55E-07 |
| Hypothetical protein | EHI_069200 | -1.76 | 2.43E-03 | 2.65E-05 |
| Short chain dehydrogenase family protein | EHI_165070 | -1.76 | 2.90E-04 | 1.68E-06 |
| Hypothetical protein | EHI_068260 | -1.75 | 9.29E-06 | 1.00E-08 |
| Hypothetical protein | EHI_096360 | -1.75 | 1.32E-04 | 5.85E-07 |
| Hypothetical protein, conserved | EHI_014070 | -1.75 | 1.73E-05 | 2.86E-08 |
| Hypothetical protein | EHI_152800 | -1.74 | 9.29E-06 | 9.70E-09 |
| Apyrase, putative | EHI_103690 | -1.72 | 9.29E-06 | 9.52E-09 |
| Hypothetical protein | EHI_125720 | -1.72 | 4.55E-04 | 2.99E-06 |
| Hypothetical protein | EHI_180780 | -1.72 | 1.29E-02 | 2.12E-04 |
| Hypothetical protein | EHI_107130 | -1.72 | 1.86E-05 | 3.28E-08 |
| Myosin 2 heavy chain, putative | EHI_014010 | -1.71 | 3.10E-05 | 7.78E-08 |
| Hpothetical protein | EHI_115280 | -1.69 | 1.84E-03 | 1.86E-05 |
| Hpothetical protein | EHI_189460 | -1.68 | 1.02E-03 | 8.77E-06 |
| Hypothetical protein | EHI_177640 | -1.67 | 3.25E-04 | 1.92E-06 |
| Hypothetical protein | EHI_154690 | -1.66 | 2.32E-05 | 4.89E-08 |
| Hypothetical protein | EHI_022970 | -1.65 | 4.62E-03 | 5.99E-05 |
| Hypothetical protein | EHI_188720 | -1.65 | 1.24E-04 | 5.40E-07 |
| Hypothetical protein | EHI_152510 | -1.63 | 1.58E-04 | 7.26E-07 |
| Hypothetical protein | EHI_158130 | -1.63 | 6.38E-05 | 2.18E-07 |
| Hypothetical protein | EHI_017750 | -1.63 | 1.73E-04 | 8.12E-07 |
| 40S ribosomal protein S11, putative | EHI_155410 | -1.62 | 3.76E-05 | 1.01E-07 |
| Hypothetical protein | EHI_181610 | -1.62 | 2.10E-05 | 4.22E-08 |
| Hypothetical protein | EHI_170150 | -1.62 | 1.71E-05 | 2.81E-08 |
| Hypothetical protein | EHI_012000 | -1.62 | 3.12E-03 | 3.66E-05 |
| Hypothetical protein | EHI_044220 | -1.61 | 8.81E-05 | 3.42E-07 |
| RhoGAP domain containing protein | EHI_153150 | -1.61 | 1.35E-05 | 1.80E-08 |
| Vacuolar proton ATPase subunit, putative | EHI_074020 | -1.61 | 1.78E-05 | 3.04E-08 |
| EhCP A5 EhCP5 cysteine proteinase, putative | EHI_168240 | -1.6 | 6.14E-05 | 2.04E-07 |
| Hypothetical protein | EHI_108710 | -1.6 | 1.00E-03 | 8.50E-06 |
| Hypothetical protein | EHI_168330 | -1.6 | 5.80E-05 | 1.84E-07 |
| Hypothetical protein | EHI_117580 | -1.6 | 6.20E-05 | 2.10E-07 |

| | | | | |
|---|---|---|---|---|
| AIG1 family protein, putative | EHI_176700 | -1.59 | 2.01E-05 | 3.88E-08 |
| Hypothetical protein | EHI_154450 | -1.59 | 1.63E-03 | 1.58E-05 |
| Hypothetical protein | EHI_144710 | -1.59 | 1.02E-03 | 8.77E-06 |
| Protein kinase domain containing protein | EHI_048710 | -1.59 | 4.51E-05 | 1.32E-07 |
| Translation initiation factor eIF 5A, putative (eif5A) | EHI_151810 | -1.59 | 1.44E-02 | 2.40E-04 |
| Hypothetical protein | EHI_007930 | -1.57 | 2.60E-05 | 5.92E-08 |
| Nucleoside transporter, putative | EHI_169580 | -1.57 | 5.85E-05 | 1.88E-07 |
| Hypothetical protein | EHI_156330 | -1.56 | 1.88E-04 | 9.09E-07 |
| Hypothetical protein | EHI_099780 | -1.56 | 5.25E-04 | 3.58E-06 |
| Hypothetical protein | EHI_056680 | -1.56 | 5.49E-05 | 1.72E-07 |
| Hypothetical protein | EHI_010030 | -1.56 | 5.50E-04 | 3.82E-06 |
| Hypothetical protein | EHI_069200 | -1.56 | 3.95E-04 | 2.47E-06 |
| Hypothetical protein, conserved | EHI_097630 | -1.56 | 8.01E-05 | 2.98E-07 |
| Galactose inhibitable lectin 35 kDa subunit Precursor, putative | EHI_183400 | -1.55 | 8.13E-05 | 3.08E-07 |
| Hypothetical protein | EHI_095830 | -1.55 | 6.61E-04 | 4.84E-06 |
| Hypothetical protein | EHI_031380 | -1.55 | 5.23E-05 | 1.61E-07 |
| Hypothetical protein | EHI_138470 | -1.55 | 7.10E-04 | 5.45E-06 |
| Hypothetical protein, conserved | EHI_050950 | -1.54 | 2.51E-04 | 1.37E-06 |
| Mob1/phocein family protein | EHI_065750 | -1.54 | 2.41E-04 | 1.28E-06 |
| Beta N acetylhexosaminidase, beta subunit (hexB) | EHI_007330 | -1.53 | 9.76E-03 | 1.50E-04 |
| Hypothetical protein | EHI_015010 | -1.53 | 6.62E-04 | 4.85E-06 |
| Hypothetical protein | EHI_165300 | -1.53 | 2.86E-04 | 1.62E-06 |
| Hypothetical protein | EHI_013900 | -1.53 | 1.81E-04 | 8.70E-07 |
| Hypothetical protein | EHI_146110 | -1.53 | 6.06E-05 | 2.00E-07 |
| Hypothetical protein, conserved | EHI_179920 | -1.53 | 1.36E-03 | 1.26E-05 |
| Molybdopterin cofactor sulfurase, putative | EHI_027700 | -1.53 | 2.65E-03 | 2.96E-05 |
| Gal/GalNAc lectin light subunit | EHI_148790 | -1.52 | 3.37E-04 | 2.02E-06 |
| Hypothetical protein | EHI_109920 | -1.52 | 2.47E-05 | 5.45E-08 |
| Methylene fatty acyl phospholipid synthase, putative | EHI_153710 | -1.52 | 4.24E-04 | 2.71E-06 |
| Acid sphingomyelinase like phosphodiesterase, putative | EHI_125660 | -1.51 | 4.13E-05 | 1.18E-07 |
| Amino acid transporter, putative | EHI_194200 | -1.51 | 3.44E-03 | 4.17E-05 |
| Hypothetical protein | EHI_140290 | -1.51 | 2.02E-04 | 1.01E-06 |
| Hypothetical protein, conserved | EHI_142370 | -1.51 | 9.61E-05 | 3.85E-07 |
| Serine/threonine protein phosphatase 2A catalytic subunit alpha isoform, putative | EHI_011950 | -1.51 | 1.62E-04 | 7.46E-07 |
| Hypothetical protein | EHI_164450 | -1.5 | 4.50E-05 | 1.31E-07 |
| Hypothetical protein | EHI_159520 | -1.5 | 6.71E-05 | 2.35E-07 |

| Gene | Gene ID | Fold change | BY | Rawp |
|---|---|---|---|---|
| **DNA Replication and DNA Repair** | | | | |
| Ehodc (ornithine decarboxylase) | EHI_100430 | 2.42 | 4.35E-06 | 1.78E-09 |
| Ehrad23 (RAD23) | EHI_001400 | 1.65 | 3.55E-04 | 2.16E-06 |
| Ehcwf12 (Cell cycle control protein cwf12) | EHI_187260 | 1.50 | 8.41E-05 | 3.21E-07 |
| DnaJ family | EHI_187000 | 4.68 | 7.20E-05 | 2.59E-07 |
| | EHI_151260 | 3.15 | 4.14E-05 | 1.19E-07 |
| EhPGK (Phosphoglycerate kinase) | EHI_188180 | 2.45 | 6.31E-06 | 4.60E-09 |
| EhCul1 (Cullin ) | EHI_118180 | 1.64 | 7.60E-05 | 2.80E-07 |
| **Cell cycle, cytokinesis, and chromosome partitioning** | | | | |
| Ehdusp (Dual specificity protein phosphatase ) | EHI_175490 | 3.0 | 2.11E-06 | 2.84E-10 |
| Ehsmc (Putative uncharacterized protein) | EHI_187190 | 2.9 | 5.86E-05 | 1.89E-07 |
| EhnudC (Nuclear movement protein) | EHI_023890 | 2.7 | 6.51E-05 | 2.26E-07 |
| Ehskip1 (S-phase kinase-associated protein 1A) | EHI_134960 | 1.8 | 6.88E-06 | 5.25E-09 |
| Ehcdc48 (cell division cycle protein 48) | EHI_045120 | 1.6 | 9.29E-06 | 9.95E-09 |
| EhCalm1 (Calmodulin) | EHI_100270 | 1.89 | 2.98E-05 | 7.33E-08 |
| EhHsp90 (Heat shock protein 90) | EHI_196940 | 4.08 | 5.27E-06 | 2.63E-09 |
| EhHsp70 (Heat shock protein 70) | EHI_026590 | 4. 64 | 1.52E-05 | 2.33E-08 |
| | EHI_052860 | 2.2 | 3.21E-05 | 8.37E-08 |
| EhHsp70A2 (Heat shock protein70) | EHI_192440 | 5.68 | 1.87E-04 | 9.01E-07 |
| | EHI_061640 | 4.56 | 3.85E-05 | 1.05E-07 |
| | EHI_132530 | 4.07 | 2.18E-05 | 4.43E-08 |
| | EHI_108130 | 3.17 | 3.21E-05 | 8.31E-08 |
| | EHI_132540 | 3.14 | 9.68E-05 | 3.89E-07 |
| | EHI_015390 | 2.92 | 2.64E-05 | 6.22E-08 |
| EhEDF1 (Helix-turn-helix) | EHI_09333 | 3.86 | 2.31E-05 | 4.83E-08 |
| EheIF-5A (Translation initiation factor eIF-5A) | EHI_177460 | 3.48 | 1.22E-05 | 1.54E-08 |
| | EHI_186480 | 1.59 | 1.52E-05 | 2.36E-08 |

| | | | | |
|---|---|---|---|---|
| EhUBI1 (Ubiquitin) | EHI_083410 | 3. 91 | 2.19E-06 | 4.32E-10 |
| | EHI_178340 | 2.50 | 5.55E-06 | 2.92E-09 |
| Ubiquitin-like | EHI_103510 | 2.45 | 3.72E-06 | 1.12E-09 |
| Ubiquitin putative | EHI_166800 | 1.80 | 1.37E-05 | 1.92E-08 |
| Ubiquitin ligase, putative | EHI_187180 | 1.7 | 1.38E-05 | 2.03E-08 |
| Ubiquitin conjugating enzyme family | EHI_048700 | 2.74 | 2.91E-05 | 7.12E-08 |
| | EHI_160740 | 2.58 | 8.89E-06 | 8.77E-09 |
| | EHI_070750 | 2.48 | 4.03E-06 | 1.27E-09 |
| | EHI_083560 | 1.84 | 1.71E-05 | 2.75E-08 |
| Ubiquitin-conjugating enzyme E2 6 | EHI_082280 | 1.66 | 6.47E-05 | 2.23E-07 |
| Rab family GTPase | EHI_164900 | 3.05 | 4.00E-05 | 1.12E-07 |
| | EHI_180410 | 2.83 | 4.34E-05 | 1.25E-07 |
| | EHI_114210 | 1.75 | 4.34E-05 | 1.25E-07 |
| Ras family GTPase | EHI_093760 | 2.48 | 2.11E-06 | 3.75E-10 |
| | EHI_154240 | 1.85 | 1.79E-05 | 3.09E-08 |
| EhRabP1 (Small GTPase) | EHI_137700 | 1.70 | 8.66E-05 | 3.32E-07 |
| Rho family GTPase | EHI_129750 | 1.72 | 3.21E-05 | 8.35E-08 |
| Rho GTPase activating protein | EHI_187110 | 1.53 | 1.07E-04 | 4.53E-07 |
| Ehodc1 (Ornithine decarboxylase) | EHI_100430 | 2.42 | 4.35E-06 | 1.78E-09 |
| EhTXRD1 (Thioredoxin-1) | EHI_004490 | 2.36 | 1.32E-05 | 1.73E-08 |
| EhSgt1 (Putative uncharacterized protein) | EHI_117820 | 3.44 | 3.07E-05 | 7.64E-08 |

**Supplementary table S3**. Genes involved in DNA replication and repair, cell division, cytokinesis and chromosome partitioning deregulated in EhPC4-overexpressing trophozoites.

| Gene | Nucleotide position | Oligonucleotide name | Sequence |
|---|---|---|---|
| ***Eh*sgt1** | -740 to -481 | P-sgt1S750 | 5´-TTGTGAAAATAAACCATTAT-3´ |
| | | P-sgt1AS500 | 5´-TGG TAT GTT AAT TAT TAT TA-3´ |
| | -500 to -231 | P-sgt1S500 | 5´-TAATAATAATTAACATACCA-3´ |
| | | P-sgt1AS250 | 5´-GGA AAC AAC TAG AAA AAG AA-3´ |
| | -250 to -1 | P-sgt1S250 | 5´- TTCTTTTTCTAGTTGTTTCC-3´ |
| | | P-sgt1AS1 | 5´-TAC TTA TTA GTT CTT TGC TT-3´ |
| ***Eh*odc1** | -750 to -481 | P-ordecS750 | 5´-TTTTAAAGACATTTGATATT-3´ |
| | | P-ordecAS500 | 5´-TAT GAA TTG AAC CAT ATT AA-3´ |
| | -500 to -231 | P-ordecS500 | 5´-TTAATATGGTTCAATTCATA-3´ |
| | | P-ordecAS250 | 5´-TCT TTT ATA TAA TAT TCT AA-3´ |
| | -250 to -1 | P-ordecS250 | 5´-TTAGAATATTATATAAAAGA-3´ |
| | | P-ordecAS1 | 5´-TGT CCA ATA ATT TAA TGA TG-3´ |
| ***Eh*cwf2** | -730 to -481 | P-cwf2S750 | 5´-AAATTTATATCAGAGCAATA-3´ |
| | | P-cwf2AS500 | 5´-CCA AGT GTT TGA ATT GAA TT -3´ |
| | -500 to -231 | P-cwf2S500 | 5´- AATTCAATTCAAACACTTGG-3´ |
| | | P-cwf2AS250 | 5´- TTG ACT AAT TAA TTG ATG GT-3´ |
| | -250 to -1 | P-cwf2S250 | 5´-ACCATCAATTAATTAGTCAA -3´ |
| | | P-cwf2AS1 | 5´- TGT TTG ATA AAT TAT AAG TT-3´ |
| ***Eh*dusp1** | -750 to -471 | P-dualS750 | 5´-ATCAAGATATTTTTGTGCCT- 3´ |
| | | P-dualAS500 | 5´-AAA AAT AGA ATA AAT GAA AA -3´ |
| | -500 to -241 | P-dualS500 | 5´-TTT TCA TTT ATT CTA TTT TT -3´ |
| | | P-dualAS250 | 5´-TTT CTC TTC CAT CTT AGA TT-3´ |
| | -260 to -1 | P-dualS250 | 5´-AATCTAAGATGGAAGAGAAA -3´ |
| | | P-dualAS1 | 5´-TGT TTC CCA TTA TTA TCA AT-3´ |
| ***Eh*smc** | -730 to -481 | P-smcS750 | 5´-AAATCAGGAAATCCTAAGTA- 3´ |

| | | | |
|---|---|---|---|
| | | P-smcAS500 | 5´-TAG AGG AGT GTT TAT ACC AT -3´ |
| | -500 to -241 | P-smcS500 | 5´- ATGGTATAAACACTCCTCTA-3´ |
| | | P-smcAS250 | 5´- AGC AAT AAT AAG AAT GAT CA-3´ |
| | -260 to -1 | P-smcS250 | 5´-TGATCATTCTTATTATTGCT -3´ |
| | | P-smcAS1 | 5´- GTA ATG ATG GTT TAT TTT CA-3´ |
| ***Eh*nudc** | -750 to -481 | P-nudcS750 | 5´- TCCCACAATATGACCCAAAT- 3´ |
| | | P-nudcAS500 | 5´- AAT CTC TAT AAA AAT AAT AG-3´ |
| | -500 to -241 | P-nudcS500 | 5´- CTATTATTTTTATAGAGATT-3´ |
| | | P-nudcAS250 | 5´- AAT TAT TTT TTT GTG CTT TA -3´ |
| | -260 to -1 | P-nudcS250 | 5´- TAAAGCACAAAAAAATAATT-3´ |
| | | P-nudcAS1 | 5´-AAC GCA ACG ATT TTG TTT TT-3´ |
| ***Eh*skp1** | -750 to -481 | P-Skp1S750 | 5´- TTTGAATAACACTAACACAG- 3´ |
| | | P-Skp1AS500 | 5´- GTA TGA ATA AAG CAA TTC TA-3´ |
| | -500 to -231 | P-Skp1S500 | 5´- TAGAATTGCTTTATTCATAC-3´ |
| | | P-Skp1AS250 | 5´-ATA AAA ATA TTG AAA TGA AC -3´ |
| | -250 to -1 | P-Skp1S250 | 5´-GTTCATTTCAATATTTTTAT -3´ |
| | | P-Skp1AS1 | 5´- TTA ACT TAT ATA TAA TTT GT-3´ |
| ***Eh*cdc48** | -750 to -481 | P-cdc48S750 | 5´-TTAAGAAATTCATATGTAGC - 3´ |
| | | P-cdc48AS500 | 5´- GAA CAG AGT AAC AAC ATG GT-3´ |
| | -500 to -231 | P-cdc48S500 | 5´-ACCATGTTGTTACTCTGTTC -3´ |
| | | P-cdc48AS250 | 5´- AAC AAA AGT TCA ATT ATT TT-3´ |
| | -250 to -1 | P-cdc48S250 | 5´-AAAATAATTGAACTTTTGTT -3´ |
| | | P-cdc48AS1 | 5´- ACT TGT ATC TTT TGG TTG TT-3´ |

**Supplementary Table S4**. Specific oligonucleotides used for chromatin immunoprecipitation assays.

**Video legends:**

Supplementary video 1. Real-time microscopy of pKT-3M cells undergoing cytokinesis

Supplementary video 2. Real-time microscopy of pKT-3M-EhPC4 cells undergoing cytokinesis

Supplementary video 3. Real-time microscopy of pKT-3M-EhNUDC cells undergoing cytokinesis
